# Inequities in Mental Health Care Facing Racialized Immigrant Older Adults With Mental Disorders Despite Universal Coverage: A Population-Based Study in Canada

**DOI:** 10.1093/geronb/gbad036

**Published:** 2023-02-26

**Authors:** Shen (Lamson) Lin

**Affiliations:** Department of Social and Behavioural Sciences, City University of Hong Kong, Kowloon, Hong Kong Special Administrative Region, China; Factor-Inwentash Faculty of Social Work, University of Toronto, Toronto, Ontario, Canada

**Keywords:** Geriatric psychiatry, Machine learning, Mental health treatment, Migration, Minority aging

## Abstract

**Objectives:**

Contemporary immigration scholarship has typically treated immigrants with diverse racial backgrounds as a monolithic population. Knowledge gaps remain in understanding how racial and nativity inequities in mental health care intersect and unfold in midlife and old age. This study aims to examine the joint impact of race, migration, and old age in shaping mental health treatment.

**Methods:**

Pooled data were obtained from the Canadian Community Health Survey (2015–2018) and restricted to respondents (aged ≥45 years) with mood or anxiety disorders (*n* = 9,099). Multivariable logistic regression was performed to estimate associations between race–migration nexus and past-year mental health consultations (MHC). Classification and regression tree (CART) analysis was applied to identify intersecting determinants of MHC.

**Results:**

Compared to Canadian-born Whites, racialized immigrants had greater mental health needs: poor/fair self-rated mental health (odds ratio [OR] = 2.23, 99% confidence interval [CI]: 1.67–2.99), perceived life stressful (OR = 1.49, 99% CI: 1.14–1.95), psychiatric comorbidity (OR = 1.42, 99% CI: 1.06–1.89), and unmet needs for care (OR = 2.02, 99% CI: 1.36–3.02); in sharp contrast, they were less likely to access mental health services across most indicators: overall past-year MHC (OR = 0.54, 99% CI: 0.41–0.71) and consultations with family doctors (OR = 0.67, 99% CI: 0.50–0.89), psychologists (OR = 0.54, 99% CI: 0.33–0.87), and social workers (OR = 0.37, 99% CI: 0.21–0.65), with the exception of psychiatrist visits (*p* = .324). The CART algorithm identifies three groups at risk of MHC service underuse: racialized immigrants aged ≥55 years, immigrants without high school diplomas, and linguistic minorities who were home renters.

**Discussion:**

To safeguard health care equity for medically underserved communities in Canada, multisectoral efforts need to guarantee culturally responsive mental health care, multilingual services, and affordable housing for racialized immigrant older adults with mental disorders.

One in three older adults might have suffered from a diagnosable mental health condition (Andreas et al., 2017). Although mental disorders in old age can be effectively treated with pharmacological and psychosocial interventions (Brenes et al., 2017; Cuijpers et al., 2006; Pinquart et al., 2006; Vasudev et al., 2019), only a minority of older adults with these conditions receive adequate treatments in primary and specialty care (Unützer et al., 2002; Van der Auwera et al., 2017). Previous epidemiological studies have found that only 31.1% depressed young–old (ages 65–74) and 20.9% depressed old–old (ages 75+) contact mental health professionals, compared to such rate of 46.9% among the middle-aged group in Canada (Crabb & Hunsley, 2006). Older people who are immigrants to a foreign country may be particularly susceptible to being underserved by mental health professionals in the host society (Lin, 2021) and such underutilization could not be explained by socioeconomic disparities or lower prevalence of mental disorders (Lin et al., 2020; Urbanoski et al., 2017); rather, it seems to reflect their collective experience of barriers to care, exposure to discrimination, and unfair treatments in the Canadian health care system (Sadavoy et al., 2004; Tuyisenge & Goldenberg, 2021).

Immigrants make up one fifth (22%) of the total Canadian population and their countries of origin have shifted from the Global North to the Global South (Lin, 2022a), representing diverse ethnicities, culture, and languages (Immigration, Refugees, and Citizenship Canada, 2022). Based on the 2016 census, the Philippines, India, China, and Nigeria were top four sources of recent immigrants to Canada, and assuming that such migration trends continue, it is estimated that more than half (57.9%) of all immigrants would be born in Asia by 2036 while a quarter (26.9%) of the total Canadian population (47.7 million) would be immigrants from Asia or Africa in 2041, up from 13.5% in 2016 (Statistics Canada, 2017; 2022). Although scientists have been examining the prevalence, disparities, and population patterns of mental health service utilization at the provincial and national levels in Canada (Chiu et al., 2018; Islam et al., 2018; Nwoke et al., 2020; Rivera et al., 2021; Urbanoski et al., 2017), contemporary immigration scholarship has typically examined race/ethnicity and migration/nativity status separately and, thus, homogenized distinct experiences of racialized immigrants and White immigrants as a monolithic category to compare with the native-born population in understanding health care inequity.

In the Canadian context, racialized immigrants refer to populations who are self-identified as non-Caucasian in race, born outside of Canada, and have been granted the right to live in Canada permanently by immigration authorities. The current study preferred the term “racialized” by society over other terminologies (e.g., ethnocultural/visible minority, people of color) to acknowledge “race” as a social construct and to signify “racialization” as a stratification process of unequal resource distribution based on ethnic hierarchies (Gkiouleka et al., 2018; Veenstra, 2009). Indeed, the invisibility of racialization in immigrant research has been recently criticized for its European-centric worldview (Lin & Fang, 2022; Sáenz & Manges Douglas, 2015). This could be a serious conceptual pitfall, because interlocking systems of oppressions, arising from race along with migration (i.e., racism and nationalism), may jointly undermine racialized immigrants’ ability to access health-enhancing resources (Brown, 2018; Koehn et al., 2013; Lin, 2021). The dismissal of racialized participants in immigrant communities, therefore, may conceal multiple layers of disadvantage that underlie inequity between racialized immigrant minorities and the dominant privileged population in Canada (Khan et al., 2017). As such, the intersectionality theory has much to offer as it unpacks various minority struggles that are often obscured within a liberal discourse of multiculturalism (Ferrer et al., 2017; Viruell-Fuentes et al., 2012).

Intersectionality, originated from Black feminist theory (Crenshaw, 1990), is a theoretical paradigm based on the notion that individuals’ numerous social positions (e.g., race, nativity) simultaneously affect human experience and health-related outcomes (Bauer et al., 2021). A burgeoning field of scholarship has adopted machining learning techniques, including decision tree methodology, to quantify intersectionality in epidemiology (Mahendran et al., 2022; Seligman et al., 2018). Decision tree is a family of data mining techniques for creating a classification system that predicts the value of a target variable by learning simple decision rules derived from data attributes (Shatte et al., 2019). It is a nonparametric supervised learning approach that has been widely applied in mental health research to identify risk factors for depression (Batterham et al., 2009; Smits et al., 2008), quality of life (D’Alisa et al., 2006), suicidal ideation (Handley et al., 2014; Niu et al., 2020), and mental health treatment-seeking (Cairney et al., 2014). Because decision tree modeling could detect meaningful intersections and split branches based on heterogeneity, this approach was considered a promising data-driven explorative tool for quantitative intersectionality research (Bauer & Scheim, 2019; Evans, 2019) and for the identification of at-risk populations in public health science (Lemon et al., 2003). Because age patterning of health inequalities is heterogeneous (Brown, 2018; Kwak, 2018), far less is known about how racial and nativity inequalities in mental health care intersect and unfold in midlife and old age. An intersectional investigation of how race, migration/nativity, and old age combine to shape mental health treatment is warranted.

Therefore, four equity-driven research questions (RQs) emerge to guide this study among older Canadians with a diagnosis of mood/anxiety disorder: Compared to the dominant group of Canadian-born (CB) Whites, are racialized immigrants (RQ1) more likely to have greater mental health needs and (RQ2) mental health consultations (MHC) with general/specialist care providers? In addition, (RQ3) what are key determinants (e.g., facilitators, barriers) of MHC and (RQ4) how do these multifaceted factors intersect with the race–migration nexus to produce inequities in mental health care? The question of “Who is being compared to whom” is critical for conceptualizing immigrant health research (Kapilashrami & Hankivsky, 2018). The decision to choose CB Whites as the reference group is based on the assumption that the intersecting power axes of race/ethnicity and migration/nativity status jointly reflect social locations of privilege in shaping the health care experience in Canada—a “White-settler society” tied to its sociocultural colonial history (Lee & Bhuyan, 2013).

## Method

### Data and Study Populations

Data were obtained from the Canadian Community Health Survey (CCHS)–Annual Components’ public-use microdata files (PUMF). The study population was derived from a combined sample of CCHS across four annual survey circles (2015–2018) via the pooled approach that has been widely applied in previous studies (Chiu et al., 2018, 2020). The resulting data set could be treated as if it is a sample from one population, based on the fact that various CCHS survey cycles were independent (Thomas & Wannell, 2009). The CCHS is an annual cross-sectional survey conducted by Statistics Canada that collects information related to health status and health care utilization among the Canadian populations aged 12 and older in all provinces and territories. A detailed explanation of the complete CCHS survey methodology and protocol can be found elsewhere (Statistics Canada, 2018). Consistent with prior research (Rivera et al., 2021), the present study only included respondents who reported having been diagnosed with a mood and/or anxiety disorder (*n* = 31,485). Because many racialized immigrants coming from low-income and middle-income countries have diverse ethnocultural perceptions of old age (Lin, 2021), the sample in this analysis was further restricted to participants aged 45 and older (*n* = 17,644). Furthermore, only respondents who participated in the “Consultations about mental health” module were included (*n* = 10,911). Those responses with missing data on key variables were excluded from the analyses and this yielded a final analytic sample of 9,099 (Lin, 2022b).

### Mental Health Needs

*Self-rated mental health (SRMH)* was assessed by asking: “In general, would you say your mental health…?” and the response was dichotomized as: 1 = good health (from “excellent” to “good”); 2 = poor health (“fair” or “poor”), consistent with previous studies (Chiu et al., 2020; Islam et al., 2014). SRMH was predictive of psychiatric conditions, self-rated health as well as perceived needs for professional help (Ahmad et al., 2014; Villatoro et al., 2018). *Self-perceived life stress* was examined by a question: “Thinking about the amount of stress in your life, would you say that most days are…?” Consistent with prior operationalization (Islam et al., 2014; Missiuna et al., 2021), it was dichotomized as: 1 = limited life stress (from “not at all stressful” to “a bit stressful”); 2 = significant life stress (from “quite a bit stressful” to “extremely stressful”). *Psychiatric comorbidity* was flagged (yes/no) when respondents reported having both an anxiety disorder and a mood disorder diagnosed by a health professional that were expected to last or had already lasted 6 months or more (Urbanoski et al., 2017). *Unmet needs for care* was defined as having “poor/fair” SRMH but reported no mental health care use in the past year among respondents with mood/anxiety disorder diagnoses (Chiu et al., 2018, 2020).

### Mental Health Consultations

Past-year prevalence of MHC (ever/never) was assessed by asking “In the past 12 months, have you seen or talked to a health professional about your emotional or mental health? (e.g., face-to-face or telephone contact).” Previous studies have found that highly distressed individuals may overreport the number of visits compared to administrative records (Rhodes & Fung, 2004). Hence, to lower the risk of recall bias, an aggregated coding (e.g., ever/never) was used for past-year MHC (Rivera et al., 2021). Frequency of visits (mean) was only presented for descriptive purpose. *Types of mental health care providers* were assessed by a follow-up question among respondents reported past-year MHC: “Whom did you see or talk to?” and chose from a list of multiple response set (0 = never; 1 = ever), including (1) family doctor/general practitioner; (2) psychiatrist; (3) psychologist; (4) nurse; (5) social worker/counselor; and (6) other professionals. While “universality” is a principle of the Canada Health Act, the accessibility of mental health services is shaped by which services are government-funded, because Canada has a two-tier mental health care system (Martin et al., 2018): public taxation (i.e., Medicare) mainly cover physician- and psychiatrist-provided services, whereas private professionals such as clinical psychologists and psychotherapists could be only financed through job-based supplemental health insurance and/or consumers’ out-of-pocket payments (Arehart-Treichel, 2005; Bartram & Stewart, 2019).

### Main Independent Variable

Race–migration nexus, based on respondents’ self-identified racial/cultural backgrounds and country of origin, was conceptualized as a key structural driver of inequalities that reflects the social stratification process of racialization and migration experiences in shaping power differentials (Lin, 2021), in which institutionalized racism and nativity-based systemic discrimination may arise (Castañeda et al., 2015; Gkiouleka et al., 2018). This intercategorical construct classified respondents into four social positionings: 1 = CB Whites (reference), 2 = CB non-Whites; 3 = foreign-born (FB) Whites; 4 = racialized immigrants (i.e., FB non-Whites). Informed by an intersectionality lens and institutional approach (Bauer, 2014), this variable was regarded as more than an individual attribute but as a product of power structures that “rank people into social hierarchies and (re) distribute social determinants of health” (Gkiouleka et al., 2018, p. 94).).

### Covariates

To reduce the possibility of spurious associations between race-migration nexus and mental health problems, potential covariates were selected based on the widely used Behavioral Model of Health Services Use (Andersen, 2008) and the Socio-Ecological Model for Older Racialized Immigrants (Lin, 2021; Lin & Fang, 2022). These covariates include *socioeconomic factors* (i.e., educational attainment, annual household income, and homeownership), *patient-side and provider-side enabling factors* (i.e., lack of a regular provider, usual source of primary care for minor health problems, marital status, primary language spoken at home, living arrangement, sense of belonging to the community), *health-need characteristics and unhealthy behaviors* (i.e., chronic disease diagnosis, current smoking status, past-year drinking habits, past-week physical activity that encapsulates sports or fitness lasting for at least 10 minutes). Detailed response options were given in Table 1. Indicators of mental health needs and mental health service use also served as covariates for each other in the statistical models.

**Table 1.** Sample Characteristics by Race–Migration Nexus and by Past-Year Mental Health Consultations (MHC), CCHS (2015–2018), Persons With Mood/Anxiety Disorders Aged ≥45 Years (*N* = 9,099)

	Full sample		By race–migration nexus					Past-year MHC	
	Unweighted *N*	Weighted all %	CB White	CB non-White	FB White	FB non-White	Chi-square	Service user	Chi-square
	*n* = 9,099	100.0%	*n* = 8,075	*n* = 95	*n* = 675	*n* = 254	Sig.	*n* = 4,419	Sig.
Mental health needs									
Self-rated mental health							<.001		<.001
Excellent/good	6,451	71.4%	72.4%	72.5%	70.2%	59.8%		48.0%	
Poor/fair	2,648	28.6%	27.6%	27.5%	29.8%	40.2%		77.1%	
Perceived life stress							.001		<.001
Not stressful	5,856	63.0%	63.3%	49.0%	65.2%	57.9%		43.6%	
Extremely/very stressful	3,243	37.0%	36.7%	51.0%	34.8%	42.1%		61.6%	
Psychiatric comorbidity							.001		<.001
Anxiety disorders Dx	2,892	31.7%	32.7%	27.5%	27.0%	26.7%		30.1%	
Mood disorders Dx	3,816	41.9%	41.2%	39.2%	49.2%	40.9%		54.4%	
Anxiety–mood disorders	2,391	26.4%	26.1%	33.3%	23.7%	32.5%		67.9%	
Unmet needs for care	847						<.001	—	—
Met: good SRMH or have MHC	8,252	91.5%	92.3%	90.2%	89.1%	85.4%		—	—
Unmet: poor/fair SRMH; no MHC	847	8.5%	7.7%	9.8%	10.9%	14.6%		—	—
Mental health care use (past year)									
Overall past-year MHC (full sample)							<.001	—	—
0 visit (never)	4,680	49.7%	48.8%	38.2%	53.4%	57.7%		—	—
1+ visits (ever)	4,419	50.3%	51.2%	61.8%	46.6%	42.3%		—	—
MHC source of providers (1 type)	3,240	36.1%	36.8%	30.4%	34.0%	30.1%	<.001	—	—
MHC source of providers (2+ types)	1,164	14.1%	14.1%	31.4%	12.6%	12.2%		—	—
Frequency of visits (mean/standard error)	2.7 (4)	—	2.7 (4)	4.1 (5)	2.4 (4)	2.5 (4)	<.001	—	—
MHC family doctor visit (yes)	2,827	33.2%	33.8%	49.0%	30.6%	26.8%	<.001	—	—
MHC psychiatrist visit (yes)	1,164	13.3%	13.0%	7.8%	13.5%	17.1%	.013	—	—
MHC psychologist visit (yes)	787	9.9%	9.9%	19.8%	10.6%	6.4%	<.001	—	—
MHC Nurse visit (yes)	232	2.6%	2.9%	1.0%	1.3%	0.5%	<.001	—	—
MHC social worker visit (yes)	770	8.0%	8.1%	17.6%	8.0%	5.3%	<.001	—	—
MHC other professionals (yes)	226	2.5%	2.5%	3.9%	3.4%	1.8%	.202	—	—
Overall past-year MHC (among poor/fair SRMH)							.004		
0 visit (never)	847	29.6%	28.0%	35.7%	36.3%	36.3%		—	—
1+ visits (ever)	1,801	70.4%	72.0%	64.3%	63.7%	63.7%		—	—
Demographics									
Age							<.001		<.001
45–54	2,753	37.7%	37.4%	39.2%	32.6%	47.9%		60.8%	
55–64	3,197	35.3%	36.3%	38.2%	30.2%	29.3%		51.4%	
65–74	2,177	19.2%	18.9%	17.6%	24.9%	16.6%		36.9%	
≥75	972	7.7%	7.4%	4.9%	12.3%	6.3%		27.2%	
Sex							.006		<.001
Male	3,090	35.6%	35.1%	42.6%	40.8%	34.8%		47.1%	
Female	6,009	64.4%	64.9%	57.4%	59.2%	65.2%		52.1%	
Socioeconomic status									
Household income							<.001		<.001
<$20k	1,971	15.7%	15.1%	19.6%	16.0%	22.9%		54.2%	
$20k to <$40k	2,187	20.3%	21.0%	12.7%	17.9%	14.6%		46.1%	
$40k to <$60k	1,561	16.8%	16.8%	26.5%	17.8%	15.1%		46.8%	
$60k to <$80k	1,022	12.4%	12.4%	15.7%	10.5%	14.8%		43.4%	
≥$80k	2,358	34.8%	34.8%	25.5%	37.8%	32.6%		55.1%	
Education							<.001		<.001
<Secondary school	1,941	18.4%	19.5%	15.7%	12.6%	12.5%		36.7%	
Secondary school	2,084	23.0%	23.5%	15.7%	20.6%	20.9%		49.3%	
Postsecondary	5,074	58.7%	57.0%	68.6%	66.8%	66.6%		54.9%	
Homeownership							.129		.003
Owned	5,797	67.5%	67.3%	64.7%	71.0%	66.0%		49.2%	
Rented	3,302	32.5%	32.7%	35.3%	29.0%	34.0%		52.5%	
Patient-side and provider-side enabling factors									
Language spoken at home							<.001		<.001
English and/or French	8,941	95.8%	99.4%	99.0%	85.8%	63.5%		50.9%	
Other home-spoken languages	158	4.2%	0.6%	1.0%	14.2%	36.5%		36.1%	
Sense of community belonging							.054		<.001
Strong	5,134	55.2%	54.7%	46.5%	57.4%	59.1%		48.0%	
Weak	3,832	43.4%	43.8%	53.5%	41.3%	39.0%		53.2%	
Not stated	133	1.5%	1.5%	0.0%	1.3%	1.8%		50.0%	
Relationship							<.001		<.001
Married	4,044	56.3%	55.8%	38.6%	60.8%	60.4%		46.8%	
Widow	3,352	27.6%	27.3%	28.7%	27.2%	30.4%		54.1%	
Single	1,703	16.1%	16.9%	32.7%	12.0%	9.2%		55.9%	
Living patterns							<.001		.002
Living alone	4,366	30.6%	31.0%	45.1%	33.1%	20.3%		52.8%	
Living with family	4,285	60.8%	60.1%	47.1%	63.0%	69.0%		48.8%	
Other types	448	8.6%	8.9%	7.8%	3.9%	10.7%		51.9%	
Lack a regular care provider							.155		<.001
No (do not lack)	8,417	93.0%	92.8%	91.2%	93.6%	95.1%		51.3%	
Yes (lack)	682	7.0%	7.2%	8.8%	6.4%	4.9%		37.0%	
Usual place for primary care							<.001		<.001
Doctor’s office	3,692	42.0%	41.0%	40.2%	47.0%	47.2%		51.7%	
Hospital outpatient clinic	627	5.9%	6.4%	4.9%	3.2%	3.1%		43.2%	
CHC (team-based care)	1,602	17.3%	17.1%	18.6%	19.1%	17.6%		53.6%	
Walk-in clinic	1,392	18.4%	17.9%	21.6%	18.8%	23.5%		51.9%	
No place but ER	1,786	16.4%	17.6%	14.7%	12.0%	8.6%		44.0%	
Health and behaviors									
Physical condition diagnoses							<.001		.03
0 condition	1,142	15.5%	15.3%	7.8%	13.8%	21.4%		51.5%	
1 condition	1,916	22.2%	22.8%	26.5%	18.3%	18.6%		52.3%	
2 conditions	2,226	24.1%	23.3%	32.4%	28.2%	27.7%		51.6%	
≥3 conditions	3,815	38.2%	38.6%	33.3%	39.8%	32.3%		47.8%	
Past-year drinking							<.001		.243
Regular drinker	4,716	54.0%	55.6%	55.4%	60.9%	24.4%		51.1%	
Occasional drinker	1,758	18.9%	19.0%	16.8%	16.2%	21.4%		49.5%	
Not past-year drinking	2,625	27.1%	25.3%	27.7%	22.9%	54.2%		49.2%	
Current smoking status							<.001		.001
Daily smoker	2,182	23.7%	24.8%	27.7%	19.8%	15.3%		52.2%	
Occasion smoker	353	4.3%	4.6%	3.0%	3.2%	2.3%		57.6%	
Nonsmoker	6,564	71.9%	70.6%	69.3%	77.0%	82.4%		49.2%	
Past-week sports							.034		<.001
Yes	3,811	42.9%	43.0%	46.5%	45.2%	37.8%		52.7%	
No	5,288	57.1%	57.0%	53.5%	54.8%	62.2%		48.5%	

*Notes*: Number of visits were presented by mean (standard deviation) and tested via analysis of variance. Number of chronic condition Dx (diagnosis), including joint pain, asthma, chronic obstructive pulmonary disease, sleep apnea, scoliosis, fibromyalgia, arthritis, back problems, osteoporosis, high blood pressure, high blood cholesterol, heart disease, stroke, diabetes, cancer, migraine headaches. CB = Canadian-born; CCHS = Canadian Community Health Survey; CHC = community health center and/or doctor's office with a team of health care professionals; Doctor’s office = “a doctor’s office with one doctor working in a solo practice” or “with several doctors working independently”; ER = emergency room; FB = foreign-born; SMRH = self-rated mental health. A regular care provider refers to access to a health professional that respondents regularly see or talk to when need care or health advice, including a family doctor (89.4%), a medical specialist (1.5%), a nurse practitioner (1.0%), and others.

### Data Analyses

Unweighted statistics were used to describe sample characteristics, while a normalized weight was applied to produce estimates that could be generalized to the Canadian population (Lin & Fang, 2022). I adopted normalizing weights, whereby the original survey weight of each unit in the subpopulation being analyzed is divided by the mean of the survey weights for all sampled units in the subpopulation. This is a commonly accepted practice that has been reported in prior studies using Statistic Canada’s probability-based survey (Lin, 2022c). First, cross-tabulation analyses were generated by Chi-square tests (χ ^2^) using weighted percentages to compare between-group differences by four racial–nativity groups and by past-year MHC. Second, to examine associations between race–migration status with mental health needs and service use (RQ1 and RQ2), binary logistic regression analyses were performed to produce odds ratios (ORs) and confidence interval (CI) while adjusting for covariates. Sensitivity tests were performed to check the robustness of these associations if necessary. I used a stringent criterion (*p* < .01) to ascertain statistical significance to account for multiple testing in multivariable regression analyses.

Lastly, to identify the intersectionality of barriers to mental health treatment from a pool of potential risk factors (RQ3), Classification and regression tree (CART) was performed in a comparison with the traditional approach of logistic regression analysis with backward selection among respondents with poor/fair SRMH (*n* = 2,648). The CART algorithm is binary decision tree that allows: (1) identifying complex interactions between variables across the measurement space (Smits et al., 2008); and (2) identifying the strongest predictor from the root node by splitting the data into child nodes repeatedly (Lemon et al., 2003). In this study, cases without past-year MHC were assigned a value of “1,” and those with past-year MHC were assigned a value of “0.” CART splitting criterion is based on node impurity as defined by the Gini improvement measure and selects the split that has the largest difference between the impurity of the parent node and a weighted average of the impurity of the two child nodes (Mahendran et al., 2022). In other words, at each spilt, the CART model selects the variable with the closest association with the past-year MHC from all influencing factors; and eventually it displays the interactions of selected variables in the form of a tree diagram. Categories of each predictor could be merged by the algorithm if they are deemed as homogenous with respect to past-year MHC. To avoid overfitting (Kreatsoulas & Subramanian, 2018), I set the stopping rules to require each child node with a minimum sample size of *n* = 25 and branching limited to five levels. I used cross-validation with 10 sample folds to calculate misclassification risk (Lin & Fang, 2023). All analyses were performed using the SPSS software package, Version 26 (IBM Corp., Armonk, NY).

## Results

### Sample Characteristics

Table 1 summarizes social-demographic covariates stratified by four racial–nativity groups and by overall past-year MHC. The entire sample (*n* = 9,099) consisted of predominantly CB Whites (83.5%), followed by CB non-Whites (1.1%), White immigrants (8.7%), and racialized immigrants (6.7%). There were more females than males (64.4% vs 35.6%). Most variables were significantly associated with race-migration nexus (*p* < .05), except for homeownership, sense of community belonging, and lack of a regular doctor. Compared to CB Whites, racialized immigrants had a higher proportion in the categories of middle adulthood (45–54 age group: 47.9% vs 37.4%), earning <20k/year (22.9% vs 15.1%), having postsecondary education (66.6% vs 57%), spoking nonofficial languages at home (36.5% vs 0.6%), and using walk-in clinics as primary care (23.5% vs 17.9%); for health and behavior factors, racialized immigrants were more likely to have fewer chronic physical conditions (21.4% vs 15.3%), report no past-year drinking (54.2% vs 25.3%), and indicate nonsmoking status (82.4% vs 70.6%).

Table 1 also presents mental health needs and service use: 28.6% of the total sample reported poor/fair SRMH, 37% reported their perceived life stressful, 26.4% reported co-occurring mood and anxiety disorders. Notably, 8.5% reported having poor/fair SRMH but not accessing any mental health services in the past year. Around half (50.3%) had MHC in the past 12 months, with mental health-related visits primarily to family doctors (33.2%), followed by psychiatrists (13.3%), psychologists (9.9%), and social workers/counselors (8%). Specifically, these mental health indicators varied significantly (*p* < .05) by race-nativity migration: racialized immigrants had the highest rate of poor/fair SRMH (4.3%) and co-occurring disorders (32.5%); in sharp contrast, they had the lowest percentage of overall MHC in the past year (42.3%) but the highest rate of psychiatrist visits (17.1%).

### RQ1: Inequities in Mental Health Needs

**Figure 1.**
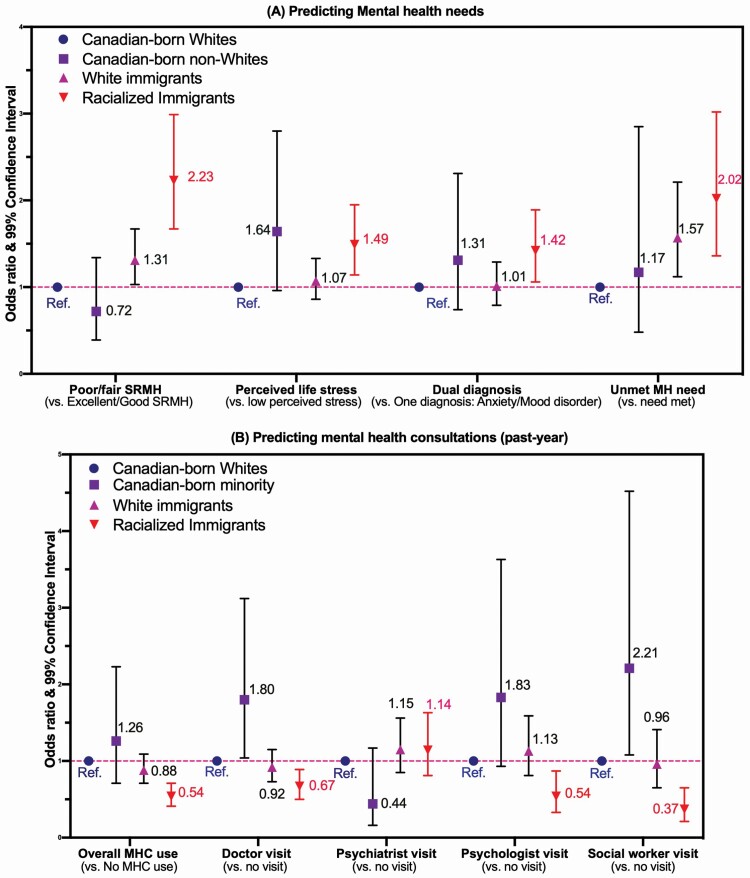
Racial–nativity inequities in (A) mental health needs and (B) mental health consultations (MHC), CCHS (2015–2018), persons with mood/anxiety disorders aged ≥45 years (*N* = 9,099). CCHS = Canadian Community Health Survey; SRMH = self-rated mental health; overall MHC = overall past-year metal health consultation. Unmet need = poor/fair SRMH but no MHC. Odds ratios were based on full adjustment (full statistics; Supplements 1 and 2) controlling for 15 factors (i.e., age, sex, family total income, education, homeownership, primary language at home, relationship, living pattern, access to regular doctor, usual place for primary care, sense of community belonging, physical condition diagnosis, drinking, smoking, and sports). See Supplement 1 for full statistics. Estimates for Canadian-born minority should be interpreted with cautions due to small sample size (*N* = 95).

Figure 1A displays the results of the logistic regression on mental health needs after controlling for covariates (full statistics; see Supplement 1). Racialized immigrants had greater needs across four indicators, including higher odds of poor/fair SRMH (Model A: adjusted odd ratio [AOR] = 2.23, 99% CI: 1.67–2.99), stressful life (Model B: AOR = 1.49, 99% CI: 1.14–1.95), psychiatric comorbidity (Model C: AOR = 1.42, 99% CI: 1.06–1.89), and unmet needs for care (Model D: AOR = 2.02, 99% CI: 1.36–3.02), compared to CB Whites. These associations persisted even after controlling for various risk factors (see Supplement 1 for full statistics), including living in low-income households (AORs range: 1.32–2.30), being a renter (AOR = 1.17), having a weak sense of belonging (AORs range: 1.31–2.82), being single/widow relationship (AORs range: 1.29–1.50), lacking of a regular doctor (AOR = 1.46), using the emergency room as primary care source (AOR = 1.26), having physical multimorbidity (AORs range: 1.33–2.03), and smoking (AORs range: 1.20–1.40), as well as all service use factors: past-year MHC (AORs range: 1.45–2.34), doctor visits (AOR = 1.23), psychiatrist visits (AORs range: 1.90–2.26), and social worker visits (AORs range: 1.34–1.45). Moreover, FB Whites were more likely than CB Whites to report poor/fair SRMH (AOR = 1.31, 99% CI: 1.03–1.67) and unmet needs (AOR = 1.57, 99% CI: 1.03–2.21).

### RQ2: Inequities in MHC and Provider Types

Figure 1B indicates results of logistic regression on mental health care use after adjusting for covariates (full statistics; see Supplement 2). Racialized immigrants had lower odds of mental health care use across most indicators, including decreased likelihood of overall past-year MHC (AOR = 0.54, 99% CI: 0.41–0.71), consultations with family doctors (AOR = 0.67, 99% CI: 0.50–0.89), psychologists (AOR = 0.54, 99% CI: 0.33–0.87), and social workers (AOR = 0.37, 99% CI: 0.21–0.65), with the exception of psychiatrist visits (*p* = .324), compared to CB Whites. These observed trends of underutilization among racialized immigrants were persistent (see Supplement 2 for full statistics), regardless of the inclusion of significant barriers to MHC (e.g., older age [AORs range: 0.11–0.73], lower household income [AORs range: 0.49–0.80], lower educational attainment [AORs range: 0.37–0.79], lack of a regular doctor [AORs range: 0.37–0.49], emergency room as primary care [AORs range: 0.67–0.71], and drinking behaviors [AORs range: 0.63–0.73]) as well as drivers to MHC (e.g., mental health needs: poor SRMH [AORs range: 1.33–2.84]; stressful life [AORs range: 1.51–1.58]; psychiatric comorbidity [AORs range: 1.23–1.49]). Interestingly, both CB non-Whites and FB Whites did not statistically differ from CB Whites in terms of overall past-year MHC, despite the fact that CB non-Whites were more likely to visit doctors (AOR = 1.80, 99% CI: 1.04–3.12) and social workers (AOR = 2.21, 99% CI: 1.08–4.52) for MHC.

Apart from the general pattern of MHC, there were marked disparities in access to diverse mental health professionals by different social positions, including social-class gradients (see Supplement 2). The income gradient was only visible in access to psychologist services (AORs range: 0.58–0.71) but not in other mental health professionals. As such, it is speculated that psychologist services (Model G) may be the major source that drove income-based disparities observed in the overall past-year MHC (Model E). Conversely, the educational gradient was still pronounced in access to almost every mental health professional, except for social workers (*p* > .05). In fact, no income or educational gap was found in consultations with social workers, suggesting that social workers were equitably accessible to socioeconomically disadvantaged communities. Speaking nonofficial language at home was associated with lower odds of consultations with family doctors (AOR = 0.63) and psychiatrists (AOR = 0.55) but with substantially greater odds of social workers visit (AOR = 2.52).

**Figure 2.**
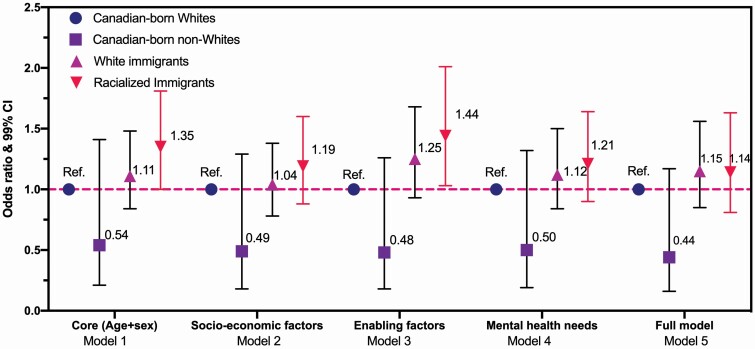
Stepwise models: the attenuating effect of mental health needs on the relation between psychiatrist visit and race–migration nexus, CCHS (2015–2018), persons with mood/anxiety disorders aged ≥45 years (*N* = 9,099). CCHS = Canadian Community Health Survey; SMRH = self-rated mental health. Estimates for Canadian-born minority should be interpreted with cautions due to small sample size (*N* = 95). To account for multiple testing, a significance level of .01 (*p* < .01) was considered statistically significant (bolded) and 99% confidence intervals (99% CIs) were used (full statistics; Supplement 3). Model 1 (core model): age + sex-adjusted. Model 2: core + socioeconomic factors (household income, education, home ownership). Model 3: core + enabling factors (primary language at home, relationship, living pattern, sense of community belonging, access to regular doctor, usual place for primary care, physical condition diagnosis, drinking, smoking, and sports). Model 4: core + mental health needs (poor SRMH, perceived life stressful, psychiatric comorbidity). Model 5: full model (core + all covariates aforementioned).

A sensitivity test was further conducted for the outcome variable of psychiatrist visits, because racialized immigrants had a higher rate of psychiatrist visits than CB Whites in the bivariate analysis (*p* = .013), a pattern which was in sharp contrast to its nonsignificant result in the latter multivariate analysis (*p* = .324). As a commonly adopted approach in public health (Akincigil et al., 2012; Zhao & Wang, 2021), a series of nested logistic regression models was estimated to further examine whether controlling for covariates explain the key association of interest (see Author Note 1). In this context, it was the linkage between race–migration nexus and psychiatrist visits (Figure 2; Supplement 3 for full statistics). Initially (Model 1), the age–sex-adjusted odds of psychiatrist visits were higher for racialized immigrants compared to CB Whites (AOR = 1.35, 99% CI: 1.00–1.81). However, this association was fully eliminated (to nonsignificant) by the inclusion of socioeconomic factors (Model 2) and mental health needs (Model 4), respectively, suggesting that the increased odds of psychiatrist visits among racialized immigrants were fully explained by differences in socioeconomic factors and mental health needs factors. Considering that racialized immigrants in this sample were more likely than CB Whites to be in the lowest household income strata (<20k/year) and have higher mental health needs (Table 1), both of which were associated with elevated odds of consultations with psychiatrists (Supplement 3), it is speculated that psychiatrists appear to provide services equitably to racialized immigrant communities in light of their heightened needs.

### RQ3: Intersecting Determinants of MHC

**Figure 3:**
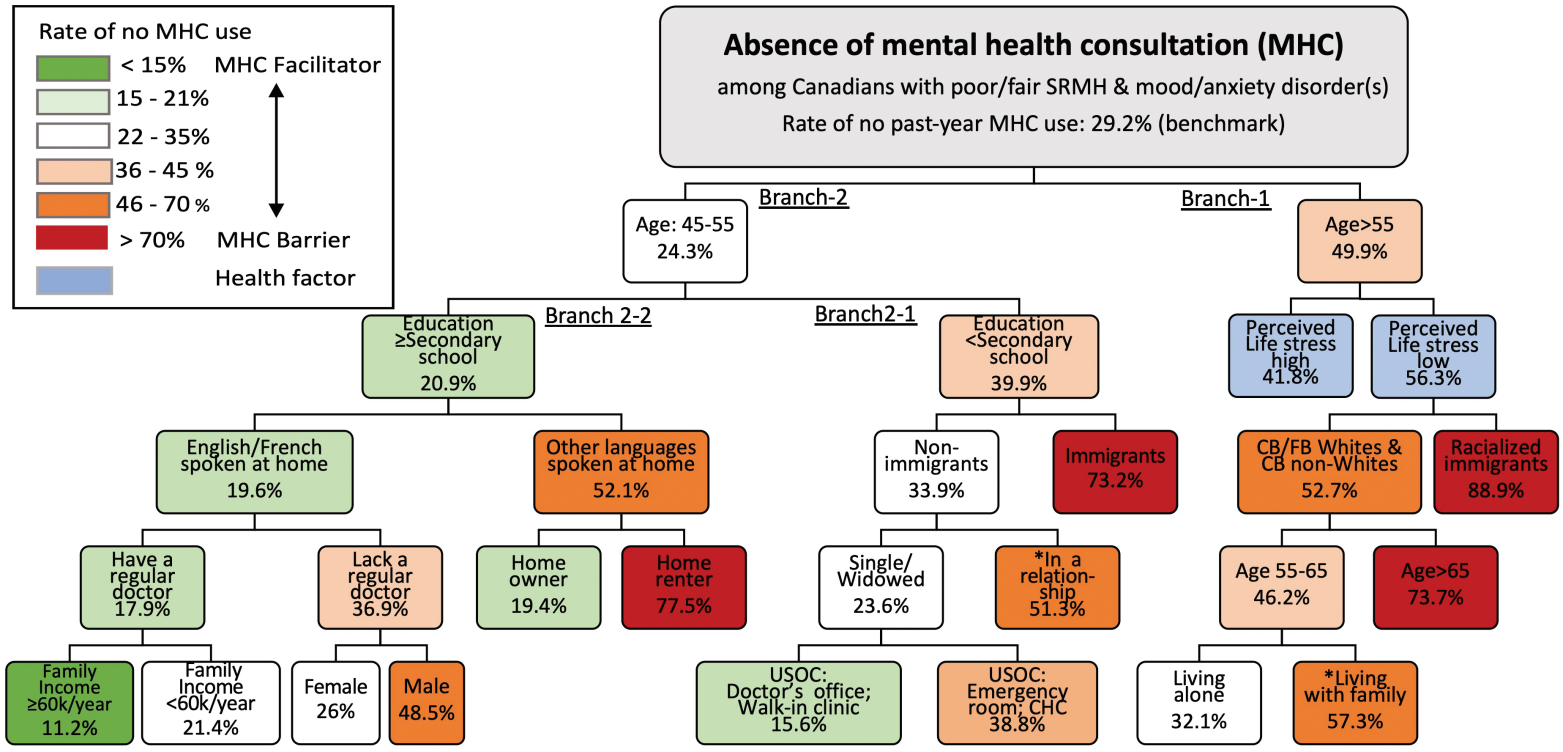
Classification and regression tree (CART) analysis: intersecting determinants of past-year MHC, CCHS (2015–2018), persons with mood/anxiety disorders and with poor/fair SRMH, aged ≥45 years (*N* = 2,648). Decision tree is based on the CART growing method to maximize within-node homogeneity (overall classification: 75.3%; sensitivity: 92%; specificity: 34.8%). Categories of each predictor could be merged by the algorithm if they are deemed as homogenous with respect to past-year MHC. CART = classification and regression tree; CB = Canadian-born; CCHS = Canadian Community Health Survey; CHC = community health center; FB = foreign-born; MHC = mental health consultations; SRMH = self-rated mental health; USOC = usual source of care.

Figure 3 provides the dendrogram of the CART analysis on the absence of overall past-year MHC among respondents with mood/anxiety disorder diagnosis who reported poor/fair SRMH (benchmark rate of no MHC: 29.2%). The percentage of no MHC ranged from 11.2% to 88.9% across 15 terminal nodes. This CART model had a total classification accuracy of 75.3% (sensitivity: 92% and specificity: 34.8%) with a risk of misclassification at 0.268. The CART algorithm identified 11 out of 17 variables as barriers to mental health treatment in this subsample, including older age, male, education ≤secondary school, home renters, racialized immigrant status, nonofficial language spoken at home, lack of a regular doctor, emergency room as primary care source, living with family members, and married/in a relationship. The latter two risk indicators seem to be counterintuitive and one possible speculation is that these factors reflect the availability of informal support (from family and/or spouse) that could address individual’s mental health needs and, thus, reduce the likelihood of mental health help-seeking in the formal care sector.

Specifically, as shown in the top parent branch, older age (aged ≥55) was the most strongly associated with the absence of MHC (49.9% vs 24.3%), compared to the middle-aged group (aged 45–54). Within these two age groups (Branch 1 and 2), there was a range of intersecting risk profiles identified in the consecutive branches, among which the race–migration nexus was the second strongest predictor as indicated by the predictive importance rate (see Supplement 4). For example (in Branch 1), on the one hand, despite having mood/anxiety disorder diagnoses, among those aged ≥55 whose perceived life stress was low, racialized immigrants were more likely not to seek MHC, compared to other three racial–nativity groups (88.9% vs 52.7%); on the other hand, for those whose perceived life stress was high, there was no further splitting of that node, because there was no significant heterogeneity in the pattern of MHC. Furthermore, in Branch 2-1 that represents middle-aged adults who did not finish secondary school, the percentage of no MHC among immigrants was more than double that of nonimmigrants (73.2% vs 33.9%). In this special circumstance, the algorithm merged four categories of the race–migration nexus as a binary migration status as it detected that FB Whites and non-Whites were homogenous (and so did CB Whites and non-Whites). In Branch 2-2, for those who finished secondary school, language spoken at home became the key factor in dividing subgroups: linguistic minorities were more likely not to seek MHC compared to English/French-speaking respondents (52.1% vs 19.6%). Moreover, among linguistic minorities, home ownership was selected as a key splitting factor, because home renters had more than triple the prevalence of no MHC than homeowners (77.5% vs 19.4%).

Notably, it is also important to compare the left-hand node and the right-hand node of the whole decision tree: middle-aged English/French-speaking adults, who finished secondary school education, with a regular doctor had the least probability of being underserved (no MHC rate: 11.2%), whereas the older-aged, racialized immigrants had the largest proportion (88.9%). To summarize, the CART tree provides nuanced insights on how race–migration nexus intersects with other risk factors, such as the abovementioned three-order intersection (between age, education, migration status) and four-order intersection (between age, education, primary language spoken at home, homeownership). The CART findings correspond well to the results from multivariable logistic regression with backward elimination method (see Supplement 4), which found identical risk factors such as racialized immigrant status, older age, and lower educational level.

## Discussion

To my best knowledge, this is the first study that illustrates how multiple social determinants interact in complex ways to shape the use of mental health care for older populations. By contrasting health needs and service use, my study reveals that, in a sample of Canadians aged 45 years and older with mood/anxiety disorder diagnoses, there were clear racial–nativity inequalities whereby racialized immigrants had a lower prevalence rate of MHC, despite having greater mental health burden as evident by poor/fair SRMH, perceived life stress, and mental morbidity than CB Whites; and consequently, racialized immigrants had double the likelihood of having unmet needs for care. This observed disproportionality highlights that mental health needs among racialized older immigrants were not adequately addressed in the current Canadian mental health care system. This study makes three significant contributions to the literature on immigrant health and minority aging.

First, the findings highlight a serious equity problem that social determinants are still playing an important role in determining MHC, including the independent effects of race–migration nexus, older age, lower income, lower educational attainment, and lack of a regular doctor. Importantly, this study illuminates the joint effect of race, migration, and old age on mental health care use (Lin, 2021). The service gap experienced by racialized immigrants may reflect multiple forms of barriers to mental health treatment that are intrinsically constructed by broader sociostructural determinants (Kalich et al., 2016; Koehn et al., 2013; Lin, 2021; Wang et al., 2019). As elucidated in the current study’s sample characteristics, racialized older immigrants were more likely to speak nonofficial languages at home, to be in the lowest income bracket, and to use walk-in clinics for primary care, all of which constitute obstacles to health care access (Thomson et al., 2015). Past research has attributed cross-cultural differences in health practices to racialized immigrants service underuse within a health care system dominated by a Western biomedical paradigm (Lin, 2021; Reitmanova & Gustafson, 2009; Wang et al., 2019). Racialized immigrants may fear being stigmatized and seek traditional ways of healing such as acupuncture, herbal remedies, and other alternative therapies (Na et al., 2016), rendering Western professional treatments as the last resort (Fang, 2010).

Another contribution of the present study lies in the investigation of inequalities in access to diverse mental health professionals across service sectors. This finding shows that racialized older immigrants were underserved by family doctors, psychologists, and social workers but not psychiatrists. Because visits to primary care practitioners are often patient-initiated, the underuse of family doctors may likely be due to immigrants’ unfamiliarity with a primary care-centric system in Canada (Tieu & Konnert, 2014) where family physicians act as first-contact gatekeepers to specialists (Kirmayer et al., 2011; Pandey et al., 2021; Wang et al., 2008). In addition, this finding echoes previous qualitative studies, which revealed that immigrants rarely consult family doctors for mental health concerns, perhaps because they perceived doctors’ role as primarily dealing with physical problems. The lower probability of psychologist visits among racialized immigrants may result from the compounding effect of financial barriers (Steele et al., 2007). The sensitivity test reveals that the seemingly higher prevalence of psychiatrist visits among racialized immigrants was primarily attributable to the severity of mental health conditions. Once these conditions were adjusted for, the initially positive association was no longer statistically significant. Initial appointments with specialists including psychiatric care often require medical referrals from family doctors. Considering racialized immigrants’ lower likelihood of doctor visits but a comparable pattern of psychiatrist visits relative to Canadian-born White service users, one may speculate that racialized immigrant patients present more severe symptoms by the time they eventually receive formal mental health treatment (Chen et al., 2003; Na et al., 2016). It may reflect racialized immigrants’ coping skills that often normalize emotional response to mental suffering (Kleinman, 2004); and thus, they are less likely to recognize mild psychological symptoms that require clinical interventions (Kirmayer, 2001; Kirmayer et al., 2011).

This study is novel by adopting a data-driven machine learning approach to substantiate the utility of intersectionality theory (Bauer, 2014; Harari & Lee, 2021) and extends its application to the field of mental health care inequalities. This intersectionality-informed analysis captures the interplay between multiple social forces including race–migration nexus and old age in shaping health care inequity (Kapilashrami & Hankivsky, 2018). It is worth mentioning that both homeownership and primary language at home stood out as important predictors of MHC via decision tree modeling but not in traditional logistic regression. This comparison suggests that the influence of being home renters (e.g., housing insecurity) linguistic minorities (e.g., language barriers) and home renter as barriers to care were not independent, but rather operated through the intersecting channels of each other. Language barriers could take various forms—such as language/accent discrimination or the lack of bilingual health professionals—that could give rise to underutilization and unfair treatment (Fang, 2010; Yoo et al., 2009). Because half of linguistic minorities (58%) were racialized immigrants in the current sample, the results further suggest that homeownership could be a salient health resource for racialized immigrants to seek MHC, possibly due to higher stability of housing, financial security, and autonomy (Finnigan, 2014; Swope & Hernández, 2019).

### Limitations

The findings should be interpreted within the context of limitations. First, the CCHS survey did not specify the time frame when respondents received their mental health diagnosis (Pelletier et al., 2017); thus, it is difficult to ascertain whether respondents had ongoing conditions with current psychiatric symptoms, episodic conditions, or a history of disorder that was already resolved at the time of the survey, despite mental disorders being chronic and often recurrent. The time frame of the past-year MHC may be subject to recall bias (Bhandari & Wagner, 2006). Second, the reliance on PUMF prohibits further investigations to provide more nuances. For example, many important measurements related to the heterogeneity of immigrant communities could not be examined via PUMF to contextualize research findings, such as country of origin, admission class/purpose of migration (e.g., refugee claimants, family reunification), or ethnic compositions. Prior research had found disparities in mental health service use by these intragroup immigration characteristics (Durbin et al., 2015; Ng & Zhang, 2021). It is also unclear whether the measure of “MHC other professionals” capture the visits to religious counselors or traditional healers (e.g., herbalist, spiritualist) that could be culturally responsive for racialized or immigrant clients, as the PUMF did not disclose open-ended responses. Third, the CCHS did not collect the information of informal support for mental health problems (e.g., communications with family/relatives/friends), which often constitutes a preferred source of help among racialized immigrants such as those with collective cultural orientation (Na et al., 2016). Alternatively, measures of living arrangement and marital status were considered as rough proxies for informal support availability in this study. Fourth, the cross-sectional nature of the CCHS survey prevented inference of the causal relationships between mental health needs and service use. Some covariates (e.g., sense of community belonging) may serve as potential mediators and future research could employ path analysis to investigate casual pathways between the race–migration nexus and health care inequity. In addition, due to the small sample size and the resultant low statistical power issue, the estimates for CB non-Whites (*N* = 95) were subjective to greater variability (i.e., wider confidence intervals) and it may reduce chances of detecting a true effect for this vulnerable group. Lastly, in the CCHS merged sample (2015–2018), respondents with a mental disorder diagnosis (*n* = 9,099) differed from individuals without a diagnosis (*n* = 63,925) to the extent that they were more likely to be female (66% vs 53.1%), members of low-income household (<20k/year: 21.7% vs 9.3%), home renters (36.3% vs 22%), widow/single people (55.6% vs 41.5%), those with have chronic physical conditions (87.5% vs 76.3%), and patients attached to a regular doctor (92.5% vs 89.1%, all *p*s < .05); hence, cautions should be given when generalizing the findings to the overall population.

## Conclusion

The race–migration nexus in Canada continues to produce discrepancies in mental health needs and mental health care use among older persons with mental disorders. To sum up, these findings make policy and clinical sense in the context of interprofessional mental health care with culturally and linguistically diverse clients. The findings underscore that structurally vulnerable populations with mental health conditions, including racialized immigrants and socioeconomic disadvantage communities, are struggling to get adequate treatment for their mental health concerns in Canada. From a policy perspective, the findings illustrate that the public-funded mental health services (Medicare) delivered by safety-net providers such as family doctors and social workers have been effective in tackling socioeconomic inequities in mental health treatment. However, the remaining treatment gaps experienced by older racialized immigrants underline the importance of expanding insurance coverage to additional mental health services (e.g., psychotherapists) that are outside the narrow bracket of Medicare. Moreover, system-level changes are needed for the federal government by reallocating funding resources to alternative healing practices (e.g., religious counseling) that are responsive to immigrants’ pluralistic understanding of mental health challenges and cultural shaping of symptoms.

From a care delivery perspective, mental health professionals should respond adequately and collaboratively to racialized immigrant older adults with mental disorders who had entered the mental health care system to receive a medical diagnosis. It is not solely a matter of training clinicians to be culturally responsive in a way that incorporates ethnocultural brokerage or ethnoracial pairing to create a safe therapeutic space, but also a call for engaging with a “structural competence approach” (Bourgois et al., 2017; Metzl & Hansen, 2014) that could intervene in broader systemic conditions affecting many racialized immigrants’ choices and behaviors (Zanchetta et al., 2021). It is essential to implement upstream interventions in dealing with fundamental social causes of health/illness (Link & Phelan, 1995; Phelan & Link, 2005), such as housing insecurity (Chen et al., 2022), for older racialized immigrant clients with mental disorders. For example, policymakers and practitioners working in the immigration settlement and mental health sectors could mirror the Housing First Project, a paradigm shift in the delivery of community mental health services primarily for homeless populations with mental disorders (Aubry et al., 2015), and expand its coverage to enables racialized immigrants’ access to permanent housing by providing them long-term rental assistance in the hosting country. In the era of globalization and mass migration, mental health clinicians should embrace an anti-oppressive, anti-discriminatory approach to empower racialized immigrant patients with mental disorders in accessing health-enhancing resources equitably at later life stage (Hulko et al., 2020).

## Supplementary Material

gbad036_suppl_Supplementary_Material

## Data Availability

The public-use microdata file of the Canadian Community Health Survey is available to Canadian researchers via Statistics Canada’s Data Liberation Initiative and to international researchers by request at dli-idd@statcan.gc.ca from Statistics Canada. The public-use data are completely deidentified and publicly available with necessary suppression methods to protect confidentiality.

## Author Note

1. Because the magnitude of odds ratio is conditional on the logistic regression’s model specification, comparing odds ratios across nested models may be problematic and it is preferable to examine average marginal effects by the linear probability model approach or probability change to compare models with stepwise inclusion of covariates (Mood, 2010; Norton & Dowd, 2018).
