# Supplementary material for: Inequities in Mental Health Care Facing Racialized Immigrant Older Adults With Mental Disorders Despite Universal Coverage: A Population-Based Study in Canada
Source: J Gerontol B Psychol Sci Soc Sci. 2023 Feb 26;78(9):1555–71. doi: 10.1093/geronb/gbad036 (PMC10461535; doi:10.1093/geronb/gbad036)
Supplement: gbad036_suppl_Supplementary_Material [file gbad036_suppl_supplementary_material.docx]

**Supplementary Material**

**Supplement 1. Predicting mental health needs among respondents with mood/anxiety disorders in the CCHS (2015-2018), age ≥ 45 years (N=9,099)**

|  | **Model A** | | | |  | **Model B** | | | |  | **Model C** | | | |  | **Model D** | | | |
| --- | --- | --- | --- | --- | --- | --- | --- | --- | --- | --- | --- | --- | --- | --- | --- | --- | --- | --- | --- |
|  | **Poor/fair SRMH** | | | |  | **Perceived life stressful** | | | |  | **Psychiatric comorbidity** | | | |  | **Unmet needs for care** | | | |
|  |  | | | |  |  | | | |  | (Mood & anxiety disorders) | | | |  | (Poor SRMH; no MHC) | | | |
|  | Nagelkerke R^2^=24.2% | | | |  | Nagelkerke R^2^=11.6% | | | |  | Nagelkerke R^2^=14% | | | |  | Nagelkerke R^2^=12.5% | | | |
| Variables | **OR** | **99%CI** | | **Sig.** |  | **OR** | **99%CI** | | **Sig.** |  | **OR** | **99%CI** | | **Sig.** |  | **OR** | **99%CI** | | **Sig.** |
| **Racial-nativity status (Ref. CB White)** |  |  |  |  |  |  |  |  |  |  |  |  |  |  |  |  |  |  |  |
| CB non-White | 0.72 | 0.39 | 1.34 | 0.174 |  | 1.64 | 0.96 | 2.80 | 0.018 |  | 1.31 | 0.74 | 2.31 | 0.224 |  | 1.17 | 0.48 | 2.85 | 0.660 |
| FB White | **1.31** | **1.03** | **1.67** | **0.004** |  | 1.07 | 0.86 | 1.33 | 0.443 |  | 1.01 | 0.79 | 1.29 | 0.902 |  | **1.57** | **1.12** | **2.21** | **0.001** |
| FB non-White | **2.23** | **1.67** | **2.99** | **<.001** |  | **1.49** | **1.14** | **1.95** | **<.001** |  | **1.42** | **1.06** | **1.89** | **0.002** |  | **2.02** | **1.36** | **3.02** | **<.001** |
| **Demographics** | | | | | | | | | | | | | | | | | | | |
| **Age (Ref. ≥75)** |  |  |  | 0.000 |  |  |  |  |  |  |  |  |  |  |  |  |  |  |  |
| 45-54 | **1.94** | **1.41** | **2.66** | **<.001** |  | **1.77** | **1.36** | **2.31** | **<.001** |  | **2.30** | **1.68** | **3.16** | **<.001** |  | 1.28 | 0.99 | 1.66 | 0.012 |
| 55-64 | **1.61** | **1.18** | **2.18** | **<.001** |  | 1.19 | 0.92 | 1.53 | 0.082 |  | **1.80** | **1.32** | **2.44** | **<.001** |  | **1.41** | **1.04** | **1.92** | **0.004** |
| 65-74 | 1.26 | 0.92 | 1.74 | 0.059 |  | **0.74** | **0.57** | **0.97** | **0.004** |  | **1.39** | **1.01** | **1.91** | **0.008** |  | 1.39 | 0.92 | 2.09 | 0.040 |
| **Female (Ref. Male)** | **0.66** | **0.57** | **0.76** | **<.001** |  | 1.10 | 0.97 | 1.25 | 0.055 |  | **1.26** | **1.09** | **1.45** | **<.001** |  | **0.57** | **0.46** | **0.71** | **<.001** |
| **Socioeconomic factors** | | | | | | | | | | | | | | | | | | | |
| **Household income (Ref. ≥$80k)** |  |  |  | 0.000 |  |  |  |  |  |  |  |  |  |  |  |  |  |  |  |
| < $20k | **1.53** | **1.19** | **1.98** | **<.001** |  | 1.25 | 0.99 | 1.58 | 0.012 |  | **1.59** | **1.23** | **2.04** | **<.001** |  | **1.75** | **1.18** | **2.60** | **<.001** |
| $20k to <$40k | **1.36** | **1.10** | **1.69** | **<.001** |  | 1.16 | 0.96 | 1.41 | 0.043 |  | **1.45** | **1.17** | **1.79** | **<.001** |  | **1.58** | **1.12** | **2.24** | **0.001** |
| $40k to <$60k | **1.36** | **1.10** | **1.68** | **<.001** |  | 0.93 | 0.77 | 1.12 | 0.313 |  | 1.14 | 0.92 | 1.41 | 0.106 |  | **1.58** | **1.12** | **2.24** | **0.001** |
| $60k to <$80k | **1.32** | **1.05** | **1.66** | **0.002** |  | 1.02 | 0.83 | 1.24 | 0.829 |  | **1.63** | **1.31** | **2.03** | **<.001** |  | **2.30** | **1.62** | **3.26** | **<.001** |
| **Education (Ref. Post-secondary)** |  |  |  |  |  |  |  |  |  |  |  |  |  |  |  |  |  |  |  |
| <Secondary school | **1.47** | **1.21** | **1.77** | **<.001** |  | **0.74** | **0.62** | **0.88** | **<.001** |  | 1.19 | 0.99 | 1.43 | 0.018 |  | **2.01** | **1.57** | **2.59** | **0.000** |
| Secondary school | 1.09 | 0.92 | 1.29 | 0.175 |  | 0.87 | 0.75 | 1.01 | 0.013 |  | 1.08 | 0.92 | 1.27 | 0.210 |  | **1.31** | **1.01** | **1.69** | **0.007** |
| **Home renter (Ref. Owner)** | 0.93 | 0.79 | 1.10 | 0.265 |  | **1.17** | **1.00** | **1.36** | **0.008** |  | 1.09 | 0.93 | 1.29 | 0.165 |  | 0.91 | 0.71 | 1.17 | 0.350 |
| **Patient-side and provider-side enabling factors** | | | | | | | | | | | | | | | | | | | |
| **Other languages at home (Ref. Official)** | 1.13 | 0.78 | 1.64 | 0.387 |  | **0.68** | **0.48** | **0.97** | **0.005** |  | 0.90 | 0.62 | 1.32 | 0.484 |  | 1.49 | 0.93 | 2.39 | 0.031 |
| **Sense of belonging (Ref. strong)** |  |  |  |  |  |  |  |  |  |  |  |  |  |  |  |  |  |  |  |
| Weak | **2.16** | **1.88** | **2.47** | **<.001** |  | **1.31** | **1.16** | **1.48** | **<.001** |  | **1.34** | **1.18** | **1.53** | **<.001** |  | **1.80** | **1.46** | **2.22** | **0.000** |
| Not stated | **2.82** | **1.68** | **4.73** | **<.001** |  | **2.61** | **1.62** | **4.20** | **<.001** |  | 0.97 | 0.56 | 1.70 | 0.892 |  | 0.85 | 0.34 | 2.11 | 0.641 |
| **Relationship (Ref. Married)** |  |  |  | 0.004 |  |  |  |  |  |  |  |  |  |  |  |  |  |  |  |
| Widow | **1.29** | **1.04** | **1.60** | **0.003** |  | 0.92 | 0.75 | 1.12 | 0.273 |  | **1.30** | **1.05** | **1.61** | **0.002** |  | 1.17 | 0.84 | 1.63 | 0.236 |
| Single | **1.34** | **1.04** | **1.72** | **0.003** |  | 0.84 | 0.67 | 1.06 | 0.053 |  | 1.04 | 0.81 | 1.34 | 0.694 |  | **1.50** | **1.03** | **2.20** | **0.006** |
| **Living pattern (Ref. With family)** |  |  |  |  |  |  |  |  |  |  |  |  |  |  |  |  |  |  |  |
| Living alone | 0.93 | 0.74 | 1.16 | 0.387 |  | 1.06 | 0.86 | 1.31 | 0.461 |  | 0.83 | 0.66 | 1.04 | 0.032 |  | 0.83 | 0.59 | 1.18 | 0.177 |
| Other types | 0.93 | 0.72 | 1.21 | 0.493 |  | 1.17 | 0.93 | 1.48 | 0.074 |  | 1.05 | 0.82 | 1.35 | 0.591 |  | 0.77 | 0.52 | 1.15 | 0.093 |
| **Lack a regular doctor (Ref. No)** | 1.24 | 0.95 | 1.62 | 0.034 |  | 0.94 | 0.74 | 1.19 | 0.472 |  | 0.78 | 0.59 | 1.04 | 0.025 |  | **1.46** | **1.03** | **2.08** | **0.006** |
| **Place for primary care (Ref. CHC)** | |  |  |  |  |  |  |  |  |  |  |  |  |  |  |  |  |  |  |
| Doctor office | 1.02 | 0.84 | 1.24 | 0.781 |  | 1.03 | 0.87 | 1.22 | 0.621 |  | 0.99 | 0.83 | 1.19 | 0.917 |  | 0.82 | 0.61 | 1.10 | 0.085 |
| Hospital outpatient clinic | 0.99 | 0.71 | 1.37 | 0.939 |  | 0.93 | 0.70 | 1.25 | 0.538 |  | 1.12 | 0.82 | 1.54 | 0.352 |  | 0.85 | 0.52 | 1.39 | 0.388 |
| Walk-in clinic | 1.07 | 0.86 | 1.34 | 0.429 |  | 1.17 | 0.96 | 1.43 | 0.037 |  | 1.02 | 0.82 | 1.27 | 0.816 |  | 1.22 | 0.88 | 1.71 | 0.118 |
| Hospital ER or no usual place | 1.15 | 0.91 | 1.46 | 0.115 |  | **1.26** | **1.02** | **1.55** | **0.004** |  | 1.05 | 0.84 | 1.32 | 0.585 |  | 1.30 | 0.93 | 1.82 | 0.041 |
| **Health and behaviours** | | | | | | | | | | | | | | | | | | | |
| **Chronic physical condition (Ref. 0)** | |  |  |  |  |  |  |  |  |  |  |  |  |  |  |  |  |  |  |
| 1 condition | 0.95 | 0.75 | 1.21 | 0.602 |  | 1.12 | 0.92 | 1.36 | 0.150 |  | 1.11 | 0.88 | 1.39 | 0.255 |  | 1.19 | 0.79 | 1.79 | 0.275 |
| 2 conditions | **1.37** | **1.09** | **1.72** | **<.001** |  | 1.15 | 0.94 | 1.40 | 0.070 |  | **1.33** | **1.07** | **1.67** | **0.001** |  | 1.32 | 0.89 | 1.96 | 0.066 |
| ≥3 conditions | **1.87** | **1.50** | **2.31** | **<.001** |  | **1.54** | **1.27** | **1.85** | **<.001** |  | **1.70** | **1.38** | **2.10** | **<.001** |  | **2.03** | **1.42** | **2.92** | **0.000** |
| **Past-week sports (Ref. No)** | **0.75** | **0.65** | **0.87** | **<.001** |  | **0.87** | **0.77** | **0.99** | **0.004** |  | 0.98 | 0.86 | 1.13 | 0.747 |  | **0.76** | **0.60** | **0.95** | **0.001** |
| **Smoking (Ref. Non-smoker)** |  |  |  |  |  |  |  |  |  |  |  |  |  |  |  |  |  |  |  |
| Daily smoker | **1.40** | **1.19** | **1.64** | **<.001** |  | **1.38** | **1.19** | **1.59** | **<.001** |  | **1.20** | **1.02** | **1.40** | **0.004** |  | 1.23 | 0.97 | 1.57 | 0.025 |
| Occasion smoker | 1.29 | 0.93 | 1.78 | 0.043 |  | 1.05 | 0.78 | 1.41 | 0.690 |  | 1.03 | 0.75 | 1.43 | 0.793 |  | 0.65 | 0.37 | 1.16 | 0.055 |
| **Past-year drinking (Ref. No intake)** | |  |  |  |  |  |  |  |  |  |  |  |  |  |  |  |  |  |  |
| Regular drinker | **0.82** | **0.70** | **0.97** | **0.002** |  | 0.92 | 0.79 | 1.07 | 0.149 |  | 0.87 | 0.74 | 1.02 | 0.021 |  | **0.73** | **0.58** | **0.94** | **0.001** |
| Occasional drinker | 0.85 | 0.70 | 1.04 | 0.034 |  | 1.12 | 0.94 | 1.33 | 0.102 |  | **0.81** | **0.67** | **0.99** | **0.006** |  | 1.03 | 0.78 | 1.35 | 0.805 |
| **Mental health consultations (past year)** | | | | | | | | | | | | | | | | | | | |
| **Overall past-year MHC (Ref. No)** | **2.36** | **1.86** | **2.99** | **<.001** |  | **1.45** | **1.16** | **1.80** | **<.001** |  | **1.74** | **1.38** | **2.19** | **<.001** |  |  |  |  |  |
| **MHC Doctor visit (Ref. No)** | 1.18 | 0.96 | 1.45 | 0.042 |  | **1.23** | **1.01** | **1.49** | **0.007** |  | 1.22 | 1.00 | 1.50 | 0.011 |  |  |  |  |  |
| **MHC Psychiatrist visit (Ref. No)** | **1.47** | **1.19** | **1.82** | **<.001** |  | 0.91 | 0.74 | 1.12 | 0.232 |  | **1.90** | **1.54** | **2.33** | **<.001** |  |  |  |  |  |
| **MHC Psychologist visit (Ref. No)** | **1.56** | **1.25** | **1.94** | **<.001** |  | **1.45** | **1.18** | **1.79** | **<.001** |  | 1.21 | 0.98 | 1.50 | 0.020 |  |  |  |  |  |
| **MHC Nurse visit (Ref. No)** | 1.43 | 0.96 | 2.13 | 0.020 |  | 1.26 | 0.87 | 1.84 | 0.109 |  | 0.70 | 0.47 | 1.05 | 0.023 |  |  |  |  |  |
| **MHC Social worker visit (Ref. No)** | **1.96** | **1.55** | **2.48** | **<.001** |  | **1.34** | **1.08** | **1.68** | **0.001** |  | **1.45** | **1.15** | **1.82** | **<.001** |  |  |  |  |  |
| **MHC Other types (Ref. No)** | **1.60** | **1.09** | **2.36** | **0.002** |  | 1.05 | 0.73 | 1.53 | 0.718 |  | 0.92 | 0.62 | 1.37 | 0.601 |  |  |  |  |  |

Note: CB=Canadian-born. FB=Foreign-born. SMRH=self-rated mental health. CHC=Community health center. CCHS=Community mental health survey. To account for multiple testing, a significance level of 0.01 (p < 0.01) was considered statistically significant (bolded) and 99% confidence intervals (99% CI) were used.

**Supplement 2. Predicting past-year mental health consultation (MHC) and contacts with specific providers among respondents with mood/anxiety disorders in the CCHS (2015-2018), age ≥ 45 years (N=9,099)**

|  | **Model E** | | | |  | **Model F** | | | |  | **Model G** | | | |  | **Model H** | | | |  | **Model I** | | | |  |
| --- | --- | --- | --- | --- | --- | --- | --- | --- | --- | --- | --- | --- | --- | --- | --- | --- | --- | --- | --- | --- | --- | --- | --- | --- | --- |
|  | **Overall Past-year MHC** | | | |  | **MHC Family doctor visit** | | | |  | **MHC Psychiatrist visit** | | | |  | **MHC Psychologist visit** | | | |  | **MHC Social worker visit** | | | |  |
|  | **Nagelkerke R^2^=19.2%** | | | |  | **Nagelkerke R^2^=12.5%** | | | |  | **Nagelkerke R^2^=15.7%** | | | |  | **Nagelkerke R^2^=12%** | | | |  | **Nagelkerke R=10.8%** | | | |  |
|  | **OR** | **99%CI** | | **Sig.** |  | **OR** | **99%CI** | | **Sig.** |  | **OR** | **99%CI** | | **Sig.** |  | **OR** | **99%CI** | | **Sig.** |  | **OR** | **99%CI** | | **Sig.** |  |
| **Racial-nativity status (Ref. CB White)** | |  |  |  |  |  |  |  |  |  |  |  |  |  |  |  |  |  |  |  |  |  |  |  |  |
| CB non-White | 1.26 | 0.71 | 2.23 | 0.301 |  | **1.80** | **1.04** | **3.12** | **0.005** |  | 0.44 | 0.16 | 1.17 | 0.030 |  | 1.83 | 0.93 | 3.63 | 0.022 |  | **2.21** | **1.08** | **4.52** | **0.004** |  |
| FB White | 0.88 | 0.71 | 1.09 | 0.111 |  | 0.92 | 0.73 | 1.15 | 0.329 |  | 1.15 | 0.85 | 1.56 | 0.239 |  | 1.13 | 0.81 | 1.59 | 0.335 |  | 0.96 | 0.65 | 1.41 | 0.786 |  |
| FB non-White | **0.54** | **0.41** | **0.71** | **<.001** |  | **0.67** | **0.50** | **0.89** | **<.001** |  | 1.14 | 0.81 | 1.63 | 0.324 |  | **0.54** | **0.33** | **0.87** | **0.001** |  | **0.37** | **0.21** | **0.65** | **<.001** |  |
| **Demographics** | | | | | | | | | | | | | | | | | | | | | | | | | |
| **Age (Ref. 45-54)** |  |  |  |  |  |  |  |  |  |  |  |  |  |  |  |  |  |  |  |  |  |  |  |  |  |
| 55-64 | **0.71** | **0.62** | **0.82** | **<.001** |  | **0.73** | **0.63** | **0.84** | **<.001** |  | 0.93 | 0.77 | 1.13 | **0.350** |  | 0.84 | 0.68 | 1.04 | 0.038 |  | **0.54** | **0.43** | **0.69** | **<.001** |  |
| 65-74 | **0.46** | **0.38** | **0.55** | **<.001** |  | **0.56** | **0.46** | **0.67** | **<.001** |  | **0.52** | **0.39** | **0.69** | **<.001** |  | **0.52** | **0.37** | **0.71** | **<.001** |  | **0.35** | **0.24** | **0.51** | **<.001** |  |
| ≥75 | **0.28** | **0.22** | **0.37** | **<.001** |  | **0.37** | **0.27** | **0.50** | **<.001** |  | **0.50** | **0.32** | **0.77** | **<.001** |  | **0.11** | **0.04** | **0.26** | **<.001** |  | **0.18** | **0.09** | **0.36** | **<.001** |  |
| **Female (Ref. Male)** | **1.23** | **1.09** | **1.40** | **<.001** |  | **1.36** | **1.19** | **1.55** | **<.001** |  | **0.83** | **0.70** | **0.99** | **0.008** |  | 1.03 | 0.84 | 1.26 | 0.737 |  | 1.09 | 0.87 | 1.36 | 0.349 |  |
| **Socioeconomic factors** | | | | | | | | | | | | | | | | | | | | | | | | | |
| **Household income (Ref. ≥$80k)** |  |  |  |  |  |  |  |  |  |  |  |  |  |  |  |  |  |  |  |  |  |  |  |  |  |
| < $20k | 0.83 | 0.66 | 1.05 | 0.039 |  | 0.94 | 0.74 | 1.19 | 0.483 |  | **1.40** | **1.02** | **1.92** | **0.006** |  | **0.58** | **0.40** | **0.85** | **<.001** |  | 1.27 | 0.87 | 1.87 | 0.109 |  |
| $20k to <$40k | **0.75** | **0.62** | **0.91** | **<.001** |  | 0.88 | 0.72 | 1.08 | 0.106 |  | 0.98 | 0.74 | 1.30 | 0.880 |  | **0.49** | **0.35** | **0.69** | **<.001** |  | 0.87 | 0.62 | 1.23 | 0.307 |  |
| $40k to <$60k | **0.80** | **0.66** | **0.96** | **0.002** |  | 0.91 | 0.75 | 1.10 | 0.177 |  | 0.96 | 0.73 | 1.27 | 0.723 |  | **0.71** | **0.52** | **0.96** | **0.003** |  | 0.73 | 0.52 | 1.04 | 0.022 |  |
| $60k to <$80k | **0.60** | **0.49** | **0.73** | **<.001** |  | **0.73** | **0.60** | **0.90** | **<.001** |  | **0.64** | **0.46** | **0.89** | **<.001** |  | 0.76 | 0.56 | 1.04 | 0.026 |  | **0.59** | **0.40** | **0.88** | **0.001** |  |
| **Education (Ref. Post-secondary)** |  |  |  |  |  |  |  |  |  |  |  |  |  |  |  |  |  |  |  |  |  |  |  |  |  |
| <Secondary school | **0.55** | **0.46** | **0.65** | **<.001** |  | **0.64** | **0.53** | **0.77** | **<.001** |  | **0.37** | **0.28** | **0.50** | **<.001** |  | **0.43** | **0.30** | **0.61** | **<.001** |  | 1.00 | 0.73 | 1.36 | 0.994 |  |
| Secondary school | **0.79** | **0.68** | **0.92** | **<.001** |  | 0.90 | 0.77 | 1.04 | 0.059 |  | **0.79** | **0.64** | **0.97** | **0.003** |  | **0.73** | **0.57** | **0.93** | **0.001** |  | 1.00 | 0.77 | 1.29 | 0.979 |  |
| **Rent home (Ref. own home)** | 1.07 | 0.91 | 1.24 | 0.285 |  | 0.97 | 0.82 | 1.13 | 0.571 |  | 1.20 | 0.97 | 1.48 | 0.026 |  | 0.89 | 0.69 | 1.14 | 0.215 |  | 1.03 | 0.79 | 1.34 | 0.772 |  |
| **Patient-side and provider-side enabling factors** | | | | | | | | | | | | | | | | | | | | | | | | | |
| **Languages at home (Ref. Official)** | 0.75 | 0.53 | 1.06 | 0.033 |  | **0.63** | **0.43** | **0.94** | **0.003** |  | **0.55** | **0.32** | **0.92** | **0.003** |  | 0.67 | 0.34 | 1.33 | 0.134 |  | **2.52** | **1.48** | **4.31** | **<.001** |  |
| **Sense of belonging (Ref. strong)** |  |  |  |  |  |  |  |  |  |  |  |  |  |  |  |  |  |  |  |  |  |  |  |  |  |
| Weak | 1.00 | 0.88 | 1.13 | 0.950 |  | **0.86** | **0.76** | **0.97** | **0.002** |  | 1.15 | 0.96 | 1.37 | 0.041 |  | **1.30** | **1.07** | **1.58** | **0.001** |  | 1.06 | 0.85 | 1.31 | 0.494 |  |
| Not stated | 1.04 | 0.63 | 1.71 | 0.838 |  | 0.99 | 0.59 | 1.65 | 0.952 |  | **1.95** | **1.05** | **3.59** | **0.005** |  | 1.21 | 0.51 | 2.85 | 0.568 |  | 0.53 | 0.16 | 1.77 | 0.173 |  |
| **Marriage (Ref. Married)** |  |  |  |  |  |  |  |  |  |  |  |  |  |  |  |  |  |  |  |  |  |  |  |  |  |
| Widow | **1.61** | **1.31** | **1.98** | **<.001** |  | **1.24** | **1.01** | **1.52** | **0.007** |  | 1.32 | 1.00 | 1.75 | 0.010 |  | 1.14 | 0.82 | 1.58 | 0.312 |  | **1.55** | **1.12** | **2.16** | **0.001** |  |
| Single | **1.45** | **1.15** | **1.83** | **<.001** |  | 0.97 | 0.76 | 1.23 | 0.736 |  | 1.36 | 0.99 | 1.88 | 0.013 |  | 0.85 | 0.58 | 1.24 | 0.261 |  | 1.24 | 0.84 | 1.82 | 0.151 |  |
| **Living patten (Ref. with family)** |  |  |  |  |  |  |  |  |  |  |  |  |  |  |  |  |  |  |  |  |  |  |  |  |  |
| Living alone | 0.97 | 0.79 | 1.21 | 0.742 |  | 0.89 | 0.72 | 1.11 | 0.173 |  | 1.07 | 0.80 | 1.42 | 0.557 |  | **1.71** | **1.23** | **2.39** | **<.001** |  | 0.87 | 0.62 | 1.23 | 0.294 |  |
| Other types | 0.90 | 0.71 | 1.14 | 0.255 |  | 1.20 | 0.95 | 1.52 | 0.042 |  | 0.99 | 0.71 | 1.39 | 0.964 |  | 0.85 | 0.57 | 1.26 | 0.277 |  | 0.65 | 0.42 | 1.00 | 0.010 |  |
| **Lack a regular doctor (Ref. No)** | **0.49** | **0.38** | **0.62** | **<.001** |  | **0.37** | **0.28** | **0.50** | **<.001** |  | 0.81 | 0.57 | 1.15 | 0.125 |  | 0.73 | 0.48 | 1.11 | 0.055 |  | 0.80 | 0.52 | 1.24 | 0.186 |  |
| **Place for primary care (Ref. CHC)** | |  |  |  |  |  |  |  |  |  |  |  |  |  |  |  |  |  |  |  |  |  |  |  |  |
| Doctor office | 0.92 | 0.78 | 1.09 | 0.207 |  | 1.11 | 0.94 | 1.32 | 0.103 |  | 0.90 | 0.71 | 1.14 | 0.237 |  | 0.97 | 0.74 | 1.26 | 0.742 |  | 0.78 | 0.59 | 1.04 | 0.024 |  |
| Hospital outpatient clinic | **0.72** | **0.54** | **0.96** | **0.003** |  | **0.61** | **0.44** | **0.85** | **<.001** |  | 0.72 | 0.47 | 1.11 | 0.053 |  | 1.06 | 0.67 | 1.70 | 0.732 |  | 1.06 | 0.67 | 1.68 | 0.756 |  |
| Walk-in clinic | 0.87 | 0.72 | 1.06 | 0.068 |  | 1.03 | 0.84 | 1.26 | 0.722 |  | 1.07 | 0.82 | 1.41 | 0.512 |  | 1.10 | 0.81 | 1.48 | 0.443 |  | **0.69** | **0.49** | **0.97** | **0.005** |  |
| Hospital ER or no usual place | **0.71** | **0.58** | **0.87** | **<.001** |  | 0.86 | 0.70 | 1.07 | 0.082 |  | **0.67** | **0.49** | **0.91** | **0.001** |  | 0.82 | 0.58 | 1.15 | 0.127 |  | 0.82 | 0.58 | 1.16 | 0.142 |  |
| **Health and behaviours** | | | | | | | | | | | | | | | | | | | | | | | | | |
| **Physical condition (Ref. 0)** |  |  |  |  |  |  |  |  |  |  |  |  |  |  |  |  |  |  |  |  |  |  |  |  |  |
| 1 condition | 1.04 | 0.85 | 1.26 | 0.640 |  | **1.23** | **1.00** | **1.50** | **0.009** |  | 0.79 | 0.60 | 1.05 | 0.034 |  | 1.13 | 0.84 | 1.53 | 0.287 |  | 1.39 | 0.98 | 1.96 | 0.014 |  |
| 2 conditions | 1.08 | 0.89 | 1.31 | 0.317 |  | **1.24** | **1.02** | **1.52** | **0.005** |  | 0.80 | 0.60 | 1.05 | 0.033 |  | 1.06 | 0.78 | 1.45 | 0.622 |  | 1.25 | 0.88 | 1.77 | 0.109 |  |
| ≥3 conditions | 0.96 | 0.80 | 1.16 | 0.572 |  | 1.06 | 0.87 | 1.28 | 0.471 |  | 0.95 | 0.73 | 1.23 | 0.605 |  | 1.17 | 0.87 | 1.57 | 0.178 |  | 1.32 | 0.94 | 1.84 | 0.037 |  |
| **Past-week sports (Ref. No)** | **1.17** | **1.03** | **1.32** | **0.001** |  | 1.04 | 0.91 | 1.18 | 0.466 |  | 1.00 | 0.83 | 1.19 | 0.972 |  | **1.48** | **1.22** | **1.81** | **<.001** |  | 1.13 | 0.91 | 1.40 | 0.153 |  |
| **Current smoking (Ref. Non)** |  |  |  |  |  |  |  |  |  |  |  |  |  |  |  |  |  |  |  |  |  |  |  |  |  |
| Daily smoker | 0.92 | 0.79 | 1.07 | 0.138 |  | 1.08 | 0.93 | 1.26 | 0.175 |  | 0.82 | 0.66 | 1.01 | 0.013 |  | 0.89 | 0.70 | 1.13 | 0.224 |  | **0.76** | **0.58** | **0.98** | **0.006** |  |
| Occasion smoker | 1.22 | 0.91 | 1.65 | 0.086 |  | **0.66** | **0.48** | **0.91** | **0.001** |  | **1.51** | **1.04** | **2.19** | **0.004** |  | 0.60 | 0.35 | 1.02 | 0.013 |  | 1.51 | 0.99 | 2.29 | 0.011 |  |
| **Past-year drinking (Ref. No intake)** | |  |  |  |  |  |  |  |  |  |  |  |  |  |  |  |  |  |  |  |  |  |  |  |  |
| Regular drinker | 0.86 | 0.74 | 1.00 | 0.010 |  | 1.12 | 0.96 | 1.31 | 0.057 |  | **0.63** | **0.51** | **0.77** | **<.001** |  | **0.73** | **0.57** | **0.92** | **0.001** |  | 1.07 | 0.82 | 1.39 | 0.531 |  |
| Occasional drinker | 0.86 | 0.72 | 1.03 | 0.033 |  | 0.96 | 0.80 | 1.16 | 0.580 |  | **0.72** | **0.56** | **0.92** | **0.001** |  | 0.80 | 0.60 | 1.08 | 0.055 |  | 1.20 | 0.88 | 1.64 | 0.134 |  |
| **Key mental health needs** | | | | | | | | | | | | | | | | | | | | | | | | | |
| **Poor SRMH (Ref. Good)** | **2.54** | **1.95** | **3.31** | **<.001** |  | **1.33** | **1.06** | **1.68** | **0.001** |  | **2.84** | **2.21** | **3.67** | **<.001** |  | **1.83** | **1.35** | **2.48** | **<.001** |  | **1.45** | **1.04** | **2.01** | **0.004** |  |
| **Perceived life stressful (Ref. No)** | **1.57** | **1.39** | **1.78** | **<.001** |  | **1.52** | **1.33** | **1.73** | **<.001** |  | 0.92 | 0.76 | 1.10 | 0.229 |  | **1.58** | **1.29** | **1.92** | **<.001** |  | **1.51** | **1.22** | **1.88** | **<.001** |  |
| **Mental comorbidity (Ref. One)** | **1.49** | **1.39** | **1.60** | **<.001** |  | **1.28** | **1.19** | **1.37** | **<.001** |  | **1.48** | **1.35** | **1.62** | **<.001** |  | **1.23** | **1.11** | **1.37** | **<.001** |  | **1.35** | **1.21** | **1.51** | **<.001** |  |

Note: CB=Canadian-born. FB=Foreign-born. SMRH=self-rated mental health. CHC=Community health center. CCHS=Community mental health survey. ER=Emergency room. To account for multiple testing, a significance level of 0.01 (p < 0.01) was considered statistically significant (bolded) and 99% confidence intervals (99% CI) were used. The results for consultations with nurses and other health providers were not shown in Table 3 because the overall prevalence was relatively low (see Table 1, <3%) and these two variables were not linked with increased odds of any mental health need (see Table 2).

**Supplement 3. Stepwise models:The attenuating effect of mental health needs on the relation between psychiatrist visit and race-migration nexus, CCHS (2015-2018), persons with mood/anxiety disorders aged ≥ 45 years (N=9,099)**

|  | **Core: Race-nativity status** | | | |  | **Socio-economic factors** | | | |  | **Enabling factors & health** | | | |  | **Mental health needs** | | | |
| --- | --- | --- | --- | --- | --- | --- | --- | --- | --- | --- | --- | --- | --- | --- | --- | --- | --- | --- | --- |
|  | **Nagelkerke R=2.4%** | | | |  | **Nagelkerke R=6.9%** | | | |  | **Nagelkerke R=7.5%** | | | |  | **Nagelkerke R=10.1%** | | | |
|  | **OR** | **99%CI** | | **Sig.** |  | **OR** | **99%CI** | | **Sig.** |  | **OR** | **99%CI** | | **Sig.** |  | **OR** | **99%CI** | | **Sig.** |
| **Racial-nativity status (Ref. CB White)** | |  |  |  |  |  |  |  |  |  |  |  |  |  |  |  |  |  |  |
| CB non-White | 0.54 | 0.21 | 1.41 | 0.097 |  | 0.49 | 0.18 | 1.29 | 0.056 |  | 0.48 | 0.18 | 1.26 | 0.050 |  | 0.50 | 0.19 | 1.32 | 0.065 |
| FB White | 1.11 | 0.84 | 1.48 | 0.333 |  | 1.04 | 0.78 | 1.38 | 0.758 |  | 1.25 | 0.93 | 1.68 | 0.052 |  | 1.12 | 0.84 | 1.50 | 0.320 |
| FB non-White | **1.35** | **1.00** | **1.81** | **0.009** |  | 1.19 | 0.88 | 1.60 | 0.145 |  | **1.44** | **1.03** | **2.01** | **0.005** |  | 1.21 | 0.90 | 1.64 | 0.103 |
| **Age (Ref. ≥75)** |  |  |  |  |  |  |  |  |  |  |  |  |  |  |  |  |  |  |  |
| 45-54 | **2.37** | **1.60** | **3.51** | **<.001** |  | **2.14** | **1.43** | **3.21** | **<.001** |  | 0.84 | 0.70 | 1.01 | 0.015 |  | 0.96 | 0.80 | 1.15 | 0.520 |
| 55-64 | **2.21** | **1.48** | **3.28** | **<.001** |  | **1.98** | **1.32** | **2.96** | **<.001** |  | **0.40** | **0.30** | **0.52** | **<.001** |  | **0.52** | **0.40** | **0.68** | **<.001** |
| 65-74 | 1.07 | 0.69 | 1.67 | 0.677 |  | 1.02 | 0.65 | 1.58 | 0.931 |  | **0.32** | **0.21** | **0.48** | **<.001** |  | **0.51** | **0.34** | **0.76** | **<.001** |
| **Female (Ref. Male)** | **0.85** | **0.72** | **1.00** | **0.009** |  | 0.86 | 0.73 | 1.01 | 0.017 |  | **0.83** | **0.70** | **0.98** | **0.004** |  | **0.83** | **0.70** | **0.99** | **0.006** |
| **Household income (Ref. ≥$80k)** |  |  |  |  |  |  |  |  | 0.000 |  |  |  |  |  |  |  |  |  |  |
| < $20k |  |  |  |  |  | **2.16** | **1.65** | **2.81** | **<.001** |  |  |  |  |  |  |  |  |  |  |
| $20k to <$40k |  |  |  |  |  | **1.35** | **1.05** | **1.74** | **0.002** |  |  |  |  |  |  |  |  |  |  |
| $40k to <$60k |  |  |  |  |  | 1.14 | 0.88 | 1.48 | 0.197 |  |  |  |  |  |  |  |  |  |  |
| $60k to <$80k |  |  |  |  |  | 0.77 | 0.56 | 1.05 | 0.032 |  |  |  |  |  |  |  |  |  |  |
| **Education (Ref. Post-secondary)** |  |  |  |  |  |  |  |  | 0.000 |  |  |  |  |  |  |  |  |  |  |
| <Secondary school |  |  |  |  |  | **0.37** | **0.28** | **0.49** | **<.001** |  |  |  |  |  |  |  |  |  |  |
| Secondary school |  |  |  |  |  | **0.80** | **0.65** | **0.97** | **0.003** |  |  |  |  |  |  |  |  |  |  |
| **Rent home (Ref. own home)** |  |  |  |  |  | **1.41** | **1.16** | **1.71** | **<.001** |  |  |  |  |  |  |  |  |  |  |
| **Languages at home (Ref. Official)** | |  |  |  |  |  |  |  |  |  | **0.48** | **0.29** | **0.81** | **<.001** |  |  |  |  |  |
| **Sense of belonging (Ref. strong)** |  |  |  |  |  |  |  |  |  |  |  |  |  |  |  |  |  |  |  |
| Weak |  |  |  |  |  |  |  |  |  |  | **1.39** | **1.18** | **1.65** | **<.001** |  |  |  |  |  |
| Not stated |  |  |  |  |  |  |  |  |  |  | **2.19** | **1.24** | **3.89** | **<.001** |  |  |  |  |  |
| **Marriage (Ref. Married)** |  |  |  |  |  |  |  |  |  |  |  |  |  |  |  |  |  |  |  |
| Widow |  |  |  |  |  |  |  |  |  |  | **1.50** | **1.16** | **1.94** | **<.001** |  |  |  |  |  |
| Single |  |  |  |  |  |  |  |  |  |  | **1.47** | **1.10** | **1.98** | **0.001** |  |  |  |  |  |
| **Living patten (Ref. with family)** |  |  |  |  |  |  |  |  |  |  |  |  |  |  |  |  |  |  |  |
| Living alone |  |  |  |  |  |  |  |  |  |  | 1.27 | 0.98 | 1.66 | 0.018 |  |  |  |  |  |
| Other types |  |  |  |  |  |  |  |  |  |  | 1.02 | 0.74 | 1.40 | 0.873 |  |  |  |  |  |
| **Lack a regular doctor (Ref. No)** |  |  |  |  |  |  |  |  |  |  | 0.82 | 0.58 | 1.14 | 0.120 |  |  |  |  |  |
| **Place for minor problems (Ref. CHC)** | |  |  |  |  |  |  |  |  |  |  |  |  |  |  |  |  |  |  |
| Doctor office |  |  |  |  |  |  |  |  |  |  | 0.92 | 0.73 | 1.16 | 0.347 |  |  |  |  |  |
| Hospital outpatient clinic |  |  |  |  |  |  |  |  |  |  | 0.69 | 0.45 | 1.05 | 0.023 |  |  |  |  |  |
| Walk-in clinic |  |  |  |  |  |  |  |  |  |  | 1.04 | 0.80 | 1.36 | 0.688 |  |  |  |  |  |
| Hospital ED or no usual place |  |  |  |  |  |  |  |  |  |  | **0.70** | **0.52** | **0.93** | **0.001** |  |  |  |  |  |
| **Morbidities (Ref. 0 conditions)** |  |  |  |  |  |  |  |  |  |  |  |  |  |  |  |  |  |  |  |
| 1 condition |  |  |  |  |  |  |  |  |  |  | 0.79 | 0.60 | 1.04 | 0.027 |  |  |  |  |  |
| 2 conditions |  |  |  |  |  |  |  |  |  |  | 0.86 | 0.66 | 1.12 | 0.135 |  |  |  |  |  |
| ≥3 conditions |  |  |  |  |  |  |  |  |  |  | 1.08 | 0.84 | 1.38 | 0.419 |  |  |  |  |  |
| **Past-week sports (Ref. No)** |  |  |  |  |  |  |  |  |  |  | 0.98 | 0.83 | 1.17 | 0.812 |  |  |  |  |  |
| **Smoking (Ref. Non-smoker)** |  |  |  |  |  |  |  |  |  |  |  |  |  |  |  |  |  |  |  |
| Daily smoker |  |  |  |  |  |  |  |  |  |  | 0.88 | 0.72 | 1.07 | 0.087 |  |  |  |  |  |
| Occasion smoker |  |  |  |  |  |  |  |  |  |  | **1.57** | **1.11** | **2.23** | **0.001** |  |  |  |  |  |
| **Past-year drinking (Ref. No inatke)** | |  |  |  |  |  |  |  |  |  |  |  |  |  |  |  |  |  |  |
| Regular drinker |  |  |  |  |  |  |  |  |  |  | **0.60** | **0.49** | **0.72** | **<.001** |  |  |  |  |  |
| Occasional drinker |  |  |  |  |  |  |  |  |  |  | **0.64** | **0.50** | **0.81** | **<.001** |  |  |  |  |  |
| **Poor SRMH (Ref. Good)** | |  |  |  |  |  |  |  |  |  |  |  |  |  |  | **3.25** | **2.56** | **4.13** | **<.001** |
| **Perceived life stressful (Ref. No)** |  |  |  |  |  |  |  |  |  |  |  |  |  |  |  | 1.00 | 0.83 | 1.19 | 0.936 |
| **Psychiatric comorbidity (Ref. One)** | |  |  |  |  |  |  |  |  |  |  |  |  |  |  | **1.50** | **1.37** | **1.64** | **<.001** |

Note: CB=Canadian-born. FB=Foreign-born. SMRH=self-rated mental health. CHC=Community health center. CCHS=Community mental health survey. ER=Emergency room. To account for multiple testing, a significance level of 0.01 (p < 0.01) was considered statistically significant (bolded) and 99% confidence intervals (99% CI) were used.

**Supplement 4** **Key determinants of past-year overall mental health consultations among respondents who reported mood/anxiety disorders with poor/fair self-rated mental health in the CCHS (2015-2018), person age ≥ 45 years (N=2,648)**

| **Classification and regression tress (CRT) n=2519*** | | | | | | |  | **Logistics regression (Backward Elimination)** | | | | |
| --- | --- | --- | --- | --- | --- | --- | --- | --- | --- | --- | --- | --- |
|  |  | Correct classification (CC)=75.3% | | | | |  |  | R=20.7% | | | |
|  |  | Not use | | Use | | Importance |  |  | CC=74.5% | | | |
| Variables | Split Values | N | % | N | % |  |  | Variables | OR | 99%CI | | Sig. |
| Total |  | 736 | 29.20% | 1783 | 70.80% |  |  |  |  |  |  |  |
| Age | 45-55 | 496 | 24.30% | 1542 | 75.70% | 100.00% |  | **Age (Ref. 45-54)** |  |  |  |  |
|  | > 55 | 240 | 49.90% | 241 | 50.10% |  |  | 55-64 | **0.54** | **0.41** | **0.72** | **<.001** |
| Age | <= 65-74 | 85 | 46.20% | 99 | 53.80% | 100.00% |  | 65-74 | **0.32** | **0.22** | **0.46** | **<.001** |
|  | > 65-74 | 42 | 73.70% | 15 | 26.30% |  |  | ≥75 | **0.17** | **0.09** | **0.29** | **<.001** |
| **Racial-nativity status** | **Racialized immigrants** | **24** | **88.90%** | **3** | **11.10%** | **38.50%** |  | **Racial-nativity status (Ref. CB White)** | |  |  |  |
|  | Other groups | 127 | 52.70% | 114 | 47.30% |  |  | CB non-White | 0.71 | 0.22 | 2.29 | 0.454 |
| **Racial-nativity status** | **Immigrants** | **41** | **73.20%** | **15** | **26.80%** | **38.50%** |  | FB White | 0.72 | 0.48 | 1.1 | 0.047 |
|  | Non-immigrants | 105 | 33.90% | 205 | 66.10% |  |  | FB non-White | **0.58** | **0.36** | **0.91** | **0.002** |
| Education | <Secondary school | 146 | 39.90% | 220 | 60.10% | 38.30% |  | **Education (Ref. Post-secondary)** |  |  |  |  |
|  | ≥Secondary school | 350 | 20.90% | 1322 | 79.10% |  |  | <Secondary school | **0.43** | **0.32** | **0.59** | **<.001** |
|  |  |  |  |  |  |  |  | Secondary school | **0.7** | **0.52** | **0.94** | **0.002** |
| Marriage | Married | 59 | 51.30% | 56 | 48.70% | 30.10% |  | **Marriage (Ref. Married)** |  |  |  | 0.015 |
|  | Single; Widow | 46 | 23.60% | 149 | 76.40% |  |  | Widow | 1.3 | 0.98 | 1.73 | 0.019 |
|  |  |  |  |  |  |  |  | Single | 0.91 | 0.65 | 1.26 | 0.443 |
| **Home ownership** | Owned | 6 | 19.40% | 25 | 80.60% | 29.40% |  |  |  |  |  |  |
|  | **Rented** | **31** | **77.50%** | **9** | **22.50%** |  |  |  |  |  |  |  |
| **Language at home** | English and/or French | 313 | 19.60% | 1288 | 80.40% | 28.50% |  |  |  |  |  |  |
|  | **Other languages** | **37** | **52.10%** | **34** | **47.90%** |  |  |  |  |  |  |  |
| Place for primary care | CHC; ER | 26 | 38.80% | 41 | 61.20% | 24.20% |  | **Place for primary care (Ref. CHC)** |  |  |  |  |
|  | GP; Walk-in/hospital OP clinic | 20 | 15.60% | 108 | 84.40% |  |  | Doctor office | 1.15 | 0.65 | 2.03 | 0.531 |
|  |  |  |  |  |  |  |  | Hospital outpatient clinic | 0.75 | 0.53 | 1.07 | 0.036 |
|  |  |  |  |  |  |  |  | Walk-in clinic | **0.62** | **0.44** | **0.86** | **<.001** |
|  |  |  |  |  |  |  |  | Hospital ED or no usual place | **0.55** | **0.39** | **0.78** | **<.001** |
| **Lack a regular doctor** | no (have provider) | 261 | 17.90% | 1199 | 82.10% | 18.20% |  | **Lack a regular doctor (Ref. No)** | **0.53** | **0.34** | **0.83** | **<.001** |
|  | **yes (lack provider)** | **52** | **36.90%** | **89** | **63.10%** |  |  |  |  |  |  |  |
| Income | <= $60k | 205 | 21.40% | 753 | 78.60% | 17.50% |  |  |  |  |  |  |
|  | > $60k | 56 | 11.20% | 446 | 88.80% |  |  |  |  |  |  |  |
| Living pattern | Living with family | 59 | 57.30% | 44 | 42.70% | 11.10% |  |  |  |  |  |  |
|  | Living alone; other type | 26 | 32.10% | 55 | 67.90% |  |  |  |  |  |  |  |
| Perceived life stress | Not stressful | 151 | 56.30% | 117 | 43.70% | 9.50% |  | **Perceived life stressful (Ref. No)** | **1.58** | **1.23** | **2.02** | **<.001** |
|  | Perceived life stressful | 89 | 41.80% | 124 | 58.20% |  |  |  |  |  |  |  |
| Sex | Female | 19 | 26.00% | 54 | 74.00% | 6.20% |  | **Female (Ref. Male)** | **1.47** | **1.14** | **1.89** | **<.001** |
|  |  |  |  |  |  |  |  | **Sense of belonging (Ref. strong)** |  |  |  |  |
|  |  |  |  |  |  |  |  | Weak | 1.05 | 0.82 | 1.35 | 0.614 |
|  |  |  |  |  |  |  |  | Not stated | **3.31** | **1.24** | **8.85** | **0.002** |
|  |  |  |  |  |  |  |  | **Morbidities (Ref. 0 conditions)** |  |  |  |  |
|  |  |  |  |  |  |  |  | 1 condition | 0.73 | 0.45 | 1.19 | 0.099 |
|  |  |  |  |  |  |  |  | 2 conditions | 0.79 | 0.5 | 1.26 | 0.195 |
|  |  |  |  |  |  |  |  | ≥3 conditions | **0.6** | **0.39** | **0.92** | **0.002** |
|  |  |  |  |  |  |  |  | **Past-week sports (Ref. No)** | 1.24 | 0.95 | 1.62 | 0.038 |
|  |  |  |  |  |  |  |  | **Smoking (Ref. Non-smoker)** |  |  |  |  |
|  |  |  |  |  |  |  |  | Daily smoker | 0.95 | 0.72 | 1.25 | 0.628 |
|  |  |  |  |  |  |  |  | Occasion smoker | **2.49** | **1.29** | **4.8** | **<.001** |
|  |  |  |  |  |  |  |  | **Past-year drinking (Ref. No intake)** |  |  |  |  |
|  |  |  |  |  |  |  |  | Regular drinker | 1.22 | 0.91 | 1.62 | 0.083 |
|  |  |  |  |  |  |  |  | Occasional drinker | 0.75 | 0.53 | 1.05 | 0.027 |
|  |  |  |  |  |  |  |  | **Psychiatric comorbidity (Ref. One)** | **1.17** | **1.03** | **1.33** | **0.001** |

*CRT analysis total sample=2519, 10-fold cross-validation was applied to generate misclassification risk.

Note: CB=Canadian-born. FB=Foreign-born. SMRH=self-rated mental health. CHC=Community health center. CCHS=Community mental health survey. ER=Emergency room. To account for multiple testing, a significance level of 0.01 (p < 0.01) was considered statistically significant (bolded) and 99% confidence intervals (99% CI) were used.
